# Exploring metal availability in the natural niche of *Streptococcus pneumoniae* to discover potential vaccine antigens

**DOI:** 10.1080/21505594.2020.1825908

**Published:** 2020-10-05

**Authors:** Lucille F. van Beek, Kristin Surmann, H. Bart van den Berg van Saparoea, Diane Houben, Wouter S. P. Jong, Christian Hentschker, Thomas H. A. Ederveen, Elena Mitsi, Daniela M. Ferreira, Fred van Opzeeland, Christa E. van der Gaast – de Jongh, Irma Joosten, Uwe Völker, Frank Schmidt, Joen Luirink, Dimitri A. Diavatopoulos, Marien I. de Jonge

**Affiliations:** a Section Pediatric Infectious Diseases, Laboratory of Medical Immunology, Radboud Institute for Molecular Life Sciences, Nijmegen, The Netherlands; b Radboud Center for Infectious Diseases, Nijmegen, The Netherlands; c Interfaculty Institute for Genetics and Functional Genomics, University Medicine Greifswald, Greifswald, Germany; d Abera Bioscience AB, Solna, Sweden; e Center for Molecular and Biomolecular Informatics, Radboud Institute for Molecular Life Sciences, Radboud University Medical Center, Nijmegen, The Netherlands; f Liverpool School of Tropical medicine, Respiratory Infection Group, Liverpool, United Kingdom of Great Britain and Northern Ireland; g Proteomics Core, Weill Cornell Medicine-Qatar, Doha, Qatar; h Department of Molecular Microbiology, Faculty of Science, Amsterdam Institute of Molecular and Life Sciences, Vrije Universiteit Amsterdam, Amsterdam, The Netherlands

**Keywords:** *Streptococcus pneumoniae*, transition metals, nasal fluid, protein antigens, colonization, *in vivo*-mimicking

## Abstract

Nasopharyngeal colonization by *Streptococcus pneumoniae* is a prerequisite for pneumococcal transmission and disease. Current vaccines protect only against disease and colonization caused by a limited number of serotypes, consequently allowing serotype replacement and transmission. Therefore, the development of a broadly protective vaccine against colonization, transmission and disease is desired but requires a better understanding of pneumococcal adaptation to its natural niche. Hence, we measured the levels of free and protein-bound transition metals in human nasal fluid, to determine the effect of metal concentrations on the growth and proteome of *S. pneumoniae*. Pneumococci cultured in medium containing metal levels comparable to nasal fluid showed a highly distinct proteomic profile compared to standard culture conditions, including the increased abundance of nine conserved, putative surface-exposed proteins. AliA, an oligopeptide binding protein, was identified as the strongest protective antigen, demonstrated by the significantly reduced bacterial load in a murine colonization and a lethal mouse pneumonia model, highlighting its potential as vaccine antigen.

## Introduction

*Streptococcus pneumoniae*, also known as the pneumococcus, colonizes the mucosal surface of the human upper airways, typically in the absence of clinical symptoms. High-density colonization enables transmission within the human population and allows the bacterium to potentially migrate to, and infect distal sites within the human body. Consequently, *S. pneumoniae* is a leading cause of a wide range of diseases, including otitis media, pneumonia, sepsis, and meningitis. Worldwide, pneumococcal disease remains a major threat among children below the age of five and in elderly [1,2].

The introduction of pneumococcal conjugate vaccines (PCVs), containing polysaccharides derived from up to 13 serotypes, has been successful in preventing disease by most vaccine-type *S. pneumoniae* [3]. However, over 95 different serotypes have been described and the emergence of non-vaccine serotypes in pneumococcal disease underscores the need for vaccine improvement [4–7]. Novel vaccines consisting of broadly protective protein antigens that reduce colonization represent a promising approach to prevent both pneumococcal disease and transmission. Therefore, there has been a concerted effort to identify protective protein antigens [8,9].

Adaptation of the pneumococcus to its primary niche, the nasopharynx, is fundamental for its persistence within the host [10–12]. The local availability of nutrients is an important aspect of this process. Transition metals are nutrients that play an essential role in cellular viability, for instance by functioning as cofactors of enzymes and facilitating resistance to oxidative stress [13,14]. Although the host environment is generally considered to be nutrient-limiting, toxicity caused by a local increase of nutrients has been described as well [15,16]. Therefore, a balance between import and export of transition metals was shown to be crucial for the survival of *S. pneumoniae* [17]. Deletion of pneumococcal genes encoding metal binding components of transporters responsible for uptake or export of for instance iron, copper, manganese, and zinc attenuated the survival of pneumococci in *in vivo* models of both colonization and disease [13,18–21].

Despite the available literature describing the importance of transition metals for the physiology of *S. pneumoniae*, there is a significant knowledge gap regarding their availability in the human host. Although several of these elements have been measured in human blood, saliva, and nasal fluid and in the mouse nose, lungs, and bloodstream, no comprehensive analysis of the transition metal’s concentrations in the human nasal cavity has been performed [16,22–26].

Our aim is to better understand pneumococcal colonization by exploring the transition metal ion concentrations in the human nasal cavity and characterize pneumococcal proteins that are abundant under these nutrient-limited conditions to discover novel vaccine antigens. To this end, transition metal levels in nasal fluid of healthy adults were determined and used to rationally design a culture medium, mimicking the *in vivo* levels of transition metals. By investigating changes in the proteome pneumococci grown under standard or nasal-mimicking conditions, we identified a number of protein antigens that were more abundant under *in vivo*-mimicking conditions. These antigens were then evaluated for their ability to confer protection against pneumococcal colonization and disease in mice using a *Salmonella* outer membrane vesicle (OMV) delivery system. Consequently, this study (i) provides new insights into the primary niche of *S. pneumoniae*, (ii) offers the composition of a nasal-mimicking culture medium that can be used to study growth and gene expression of a variety of bacterial respiratory pathogens, and (iii) identifies pneumococcal protein antigens that may be potential vaccine candidates.

## Results

### Free transition metals in human nasal fluid are scarce

**Figure 1.**
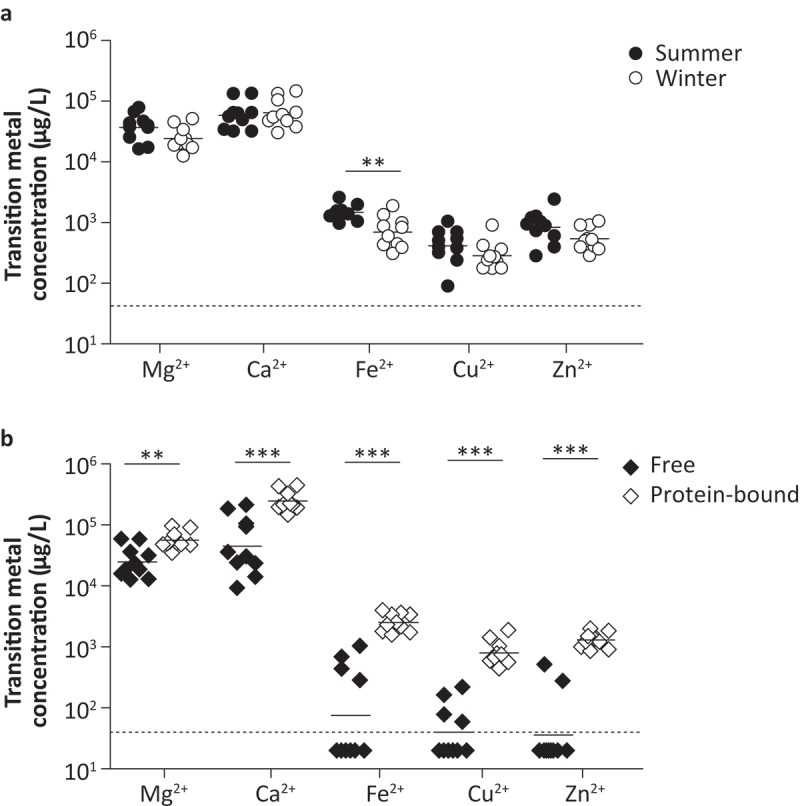
Nasal transition metal levels are highly similar between summer and winter season and mainly bound to proteins. Nasal fluid of ten healthy adults was collected from the inferior turbinate and Mg^2+^, Ca^2+^, Fe^2+^, Cu^2+^, Zn^2+^, Co^2+^, and Mn^2+^ concentrations were determined using ICP-MS. (a) Transition metal levels during summer and winter season, of which 8 out of 10 individuals participated at both time points. (b) Transition metal levels in protein depleted and protein-rich nasal fluid fractions collected during the winter season, representing free and protein-bound transition metals, respectively. Dashed lines indicate the detection limit, symbols represent individual volunteers and horizontal lines indicate the geometric mean of each group. Co^2+^ and Mn^2+^ levels were below detection limit (<40 µg/L) for all volunteers during all measurements. Statistical significance was determined using a two-tailed Mann-Whitney U test with 95% confidence intervals. ** p-value < 0.01, *** p-value < 0.001.

Nasal fluid samples were collected from 10 healthy adult volunteers to measure the transition metal composition using Induction-Coupled Plasma Mass Spectrometry (ICP-MS). Spatial differences in transition metal levels within the upper respiratory tract (URT) were examined by comparing the concentrations in nasal fluid obtained from the nasopharynx and from the inferior turbinate (Figure S1). Overall, transition metal levels were similar between these two adjacent anatomical sites, apart from zinc levels, which were significantly higher in the nasopharynx than in the inferior turbinate. In follow-up experiments, nasal fluid was collected from the inferior turbinate, as this procedure is less invasive. Nasal fluid collection was performed both during the summer (July) and the winter (January), to evaluate potential seasonal changes in metal levels (Figure 1(a)). From 8 out of 10 individuals paired samples from both seasons were available. Regardless of sampling method or season, over 10-fold higher levels of magnesium and calcium were observed, as compared to iron, copper or zinc. Of note, manganese and cobalt levels were below the detection limit (<40 µg/L) in all samples. A separate measurement on nasal fluid of 10 volunteers using a more sensitive ICP-MS instrument identified a mean manganese level of 33.8 µg/L, whereas cobalt remained below the detection limit (<1.5 µg/L). Seasonal differences were only observed for iron levels, which were higher during the summer season. Next, we assessed the levels of free and protein-bound or complexed metals by dividing nasal fluid samples into a protein-rich and protein-depleted fraction (Figure 1(b)). For this analysis nasal fluid collected in the winter season was used. Both levels of complexed and free magnesium and calcium were high, whereas iron, copper, and zinc were mainly found to be bound to proteins. These results show that for iron, copper, and zinc the freely available levels in nasal fluid are low. To study whether colonization with *S. pneumoniae* changes the transition metal levels in the human nose and whether the levels prior to the exposure to the bacterium might play a role in whether carriage will be established, we evaluated the free and protein-bound transition metal levels in nasal fluid of volunteers of the Experimental Human Pneumococcal Challenge (EHPC) model. This data suggest that transition metal levels in carriage positive and carriage negative individuals are highly similar, with no clear trend in transition metal levels during the 1st week after inoculation (Figure S2). Notably, large inter-volunteer variation as well as intra-volunteer variation over time was observed for free iron and zinc.

### Transition metal composition of culture medium impacts the pneumococcal proteome

**Figure 2.**
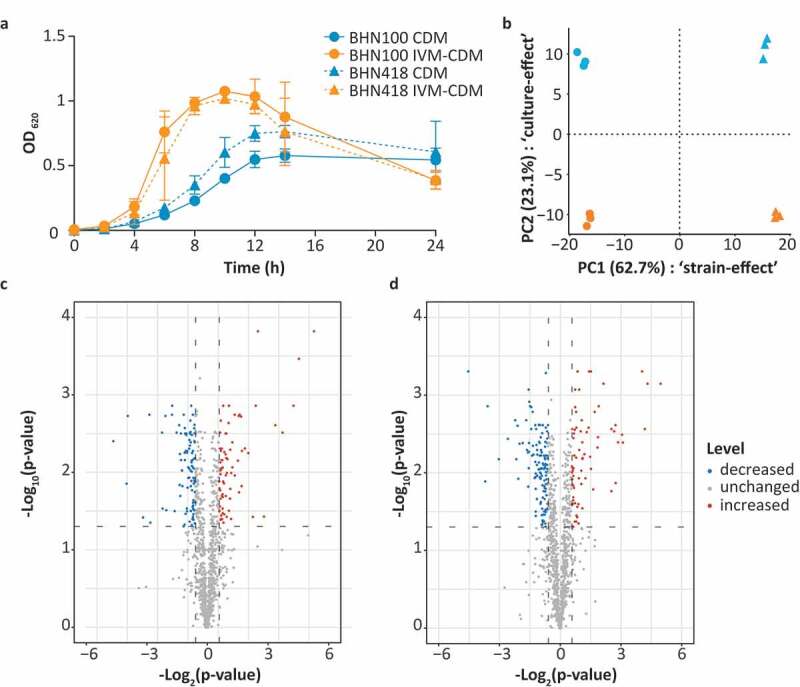
Transition metal levels impact growth kinetics and proteomic profile of *S. pneumoniae*. BHN100 (serotype 19 F) and BHN418 (serotype 6B) were passaged once in standard CDM or IVM-CDM and subsequently used for analysis of the growth kinetics (a) and the proteome (b–d). (a) The graph shows the mean optical density (OD) at 620 nm of four independent experiments, with error bars representing the standard deviation. (b) Whole cell shotgun proteomics data from logarithmic phase bacteria (OD 0.3) of three independent cultures were used for principal component analysis. Median normalized intensities of 1124 proteins were used as input. X-axis represents principal component (PC) 1 showing differences between strains, y-axis represents PC2 showing differences due to the culture conditions tested. (c,d) Volcano plots showing proteins with increased (red), decreased (blue) and unchanged (gray) levels in IVM-CDM *versus* CDM for both BHN100 (c) and BHN418 (d), based on >|1.5|-fold change in median normalized protein intensities and a q-value < 0.05.

Based on the measured metal concentrations in nasal fluid, a standard Chemically Defined Medium (CDM) was adjusted to *In Vivo*-Mimicking (IVM-CDM) conditions (Table 1). *S. pneumoniae* strains BHN100 and BHN418 were both passaged once in either CDM or IVM-CDM prior to further analysis. Clear differences in bacterial growth kinetics were seen between CDM and IVM-CDM conditions (Figure 2(a)). Pneumococci cultured in IVM-CDM showed faster growth kinetics than pneumococci cultured in CDM. Moreover, IVM-CDM cultured bacteria reached a higher OD and lysed faster than CDM-cultured pneumococci. Subsequently, we investigated differences in protein patterns between bacteria grown under these conditions with shotgun proteomics. Bacteria were harvested in the exponential growth phase and analyzed in triplicate (Table S1). In total, this approach identified 1205 proteins in at least one of the replicates of one of the conditions, and 1124 proteins were detected in all replicates per strain. Principal component analysis (PCA) on these 1124 proteins indicated that most of these differences were strain dependent (62.7%) (Figure 2(b)). Nonetheless, for both strains, more than 23% of the observed variation was related to differences in transition metal composition of the medium. In total, 75 proteins displayed significantly increased levels (>1.5-fold; q-value < 0.05) in IVM-CDM *versus* CDM in either BHN100 or BHN418 (Figure 2(c,d)). Conversely, 105 and 139 proteins had significantly decreased levels (>1.5-fold; q-value < 0.05) in IVM-CDM *versus* CDM in either BHN100 or BHN418, respectively. Furthermore, 15 proteins showed an ON/OFF pattern in at least one of the strains. This ON/OFF pattern was defined by the detection of a peptide derived from a particular protein in two out of three replicates in one of the culture conditions *versus* absence of peptides of the same protein in all three replicates of the other culture condition (Table S1). This proteomic analysis yielded 38 proteins with significantly increased levels in IVM-CDM *versus* CDM in both strains and 63 proteins with significantly decreased levels in IVM-CDM *versus* CDM in both strains (Tables S2 and S3).

**Table 1.** Transition metal concentrations in nasal fluid, standard CDM and IVM-CDM.

	Transition metal concentration (µg/L)						
	Mg^2+^	Ca^2+^	Mn^2+^	Fe^2+^	Co^2+^	Cu^2+^	Zn^2+^
CDM^a^	22,168	8,291	10,358	95	632	48	1,073
IVM-CDM^a^	30,910	83,839	<40^c^	1,232	<40^c^	336	668
Nasal fluid^b^	34,024	70,106	<40^c^	1,174	<40^c^	411	787

CDM: chemically defined medium; IVM-CDM: *in vivo*-mimicking chemically defined medium (prepared as described in Table S6 and Kloosterman et al. [79]).

Carbon source: 5 g/L Glucose.

^a^Mean transition metal levels of three independent measurements.

^b^Mean transition metal levels in undiluted nasal fluid collected from the inferior turbinate during the summer and winter season from 10 individuals at each time point.

^c^Levels were below the detection limit of 40 µg/L.

### *In vivo*-mimicking culture condition revealed potential pneumococcal protein antigens

**Figure 3.**
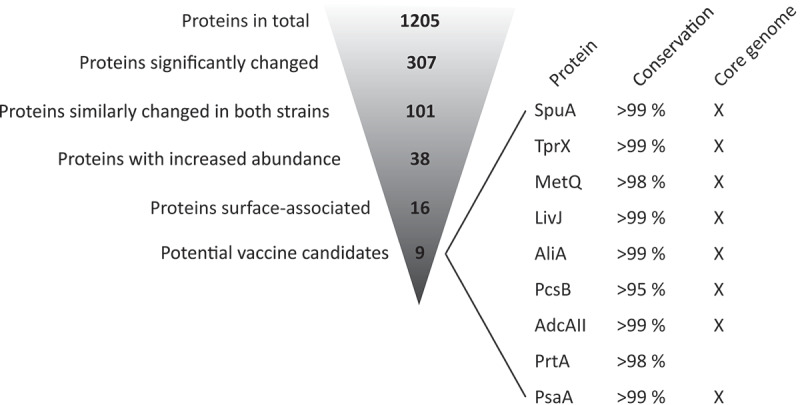
Antigen selection strategy based on comparative proteome profiling of pneumococci. Potential vaccine antigens were selected based on the proteomic datasets obtained from *S. pneumoniae* strains BHN100 and BHN418 cultured in standard CDM and IVM-CDM. In total, 1205 proteins were detected in at least one replicate of one of the strains of one of the culture conditions, see also Table S1. Of these proteins, 307 proteins were significantly different between the two culture conditions for at least one of the strains (>|1.5|-fold difference, q < 0.05 or a ON/OFF pattern). 101 proteins showed a similarly changed level in both strains, meaning that the protein level was significantly increased in both strains or decreased in both strains. Proteins with increased levels in IVM-CDM *versus* CDM (38 proteins) were selected, followed by the selection for putative surface proteins (16 proteins) and proper orientation into the bacterial membrane (9 proteins). Nine proteins of interest were checked for their amino acid conservation level based on the sequence identity using NCBI-BLAST (top 100 sequences of *S. pneumoniae* taxid:1313) [28] and presence of the encoding gene in the core genome of *S. pneumoniae* [29].

Based on the data of the bacterial proteome analyses, we selected candidate vaccine antigens for further analysis. Antigens were identified based on a statistically significant >1.5-fold increased protein level in IVM-CDM *versus* CDM in both strains, the prediction of surface association and a surface-exposed orientation as determined by PSORTb 3.0 [27]. This selection strategy yielded nine putative surface-exposed proteins: SpuA, TprX, MetQ, LivJ, AliA, PcsB, AdcAII, PrtA, and PsaA (Figure 3). For these nine proteins, we assessed the level of amino acid sequence identity as a measure of protein conservation among pneumococcal strains using the NCBI-BLAST database [28], and checked whether the encoding genes are part of the previously published core genome of *S. pneumoniae* [29]. All proteins showed a high conservation level (>95%) and for 8 out of 9 proteins the encoding genes were shown to be part of the pneumococcal core genome, suggesting that these proteins could potentially provide broad protection. Although *prtA* is not part of the core genome based on this analysis we included PrtA in our follow-up experiments, because it was found to be expressed by almost all serotypes, and recognized by 94% of the patient sera tested in a study of Wizemann et al. [30], suggesting that this protein is present in the majority of the circulating strains.

### Potential antigens discovered under in vivo-mimicking conditions are expressed and immunogenic during colonization

Next, we investigated whether the genes encoding these nine target antigens are expressed by *S. pneumoniae* during colonization of the murine and human URT. For this purpose, mice were intranasally infected with *S. pneumoniae* (1 × 10^6^ colony forming units (CFU) BHN418 in 5 µl) followed by a nasal wash 3 d later. Pneumococci colonizing the human URT were retrieved from volunteers participating in the EHPC studies (inoculated with 8 × 10^4^ CFU BHN418 per nostril) [31] using an oropharyngeal swab. Harvested bacteria were used for qPCR analysis, which confirmed the expression of all nine genes during colonization (Table S4). Overall, the highest expression levels were detected for *psaA*, followed by *metQ, pcsB*, and *aliA*. Subsequently, we evaluated whether pneumococcal colonization (1 × 10^6^ CFU BHN100 in 5 µl) in mice induces protein-specific antibodies in serum detectable 4 weeks after infection by ELISA (Figure S3). The highest IgG responses were detected against PsaA and AliA. MetQ, AdcAII, and PrtA-specific antibodies could only be measured in some of the mice. Taken together, these results confirm that all genes encoding the selected putative antigens are expressed during colonization in mice and humans, of which at least PsaA, AliA, MetQ, AdcAII, and PrtA induced antibody responses in mice.

### Immunization with outer membrane vesicles displaying potential antigens induces systemic antigen-specific antibodies, which bind to S. pneumoniae in vitro

To explore the protective efficacy of the selected proteins, all proteins were recombinantly produced, purified and coupled to lipopolysaccharide (LPS)-detoxified *Salmonella* OMVs, which we have previously used as a mucosal antigen delivery system to establish a reduction in pneumococcal colonization, while not requiring the addition of an adjuvant [32,33]. To this end, OMVs were produced displaying HbpD-SpyCatcher on the surface (OMV-HbpD-SpC, hereafter “OMV”) to allow ligation of purified antigens carrying a cognate SpyTag (SpT) sequence [34]. Coupling of antigens to OMVs was confirmed by a band shift of the HbpD-SpC-SpT-Antigen fusion protein compared to the HbpD-SpC protein, corresponding to the size of the SpT-Antigen fusion protein as determined by SDS-PAGE (Figure S4). The amount of coupled antigen per OMV dose was quantified based on densitometric analysis and ranged from 11 to 315 ng depending on the antigen (Table S5).

**Figure 4.**
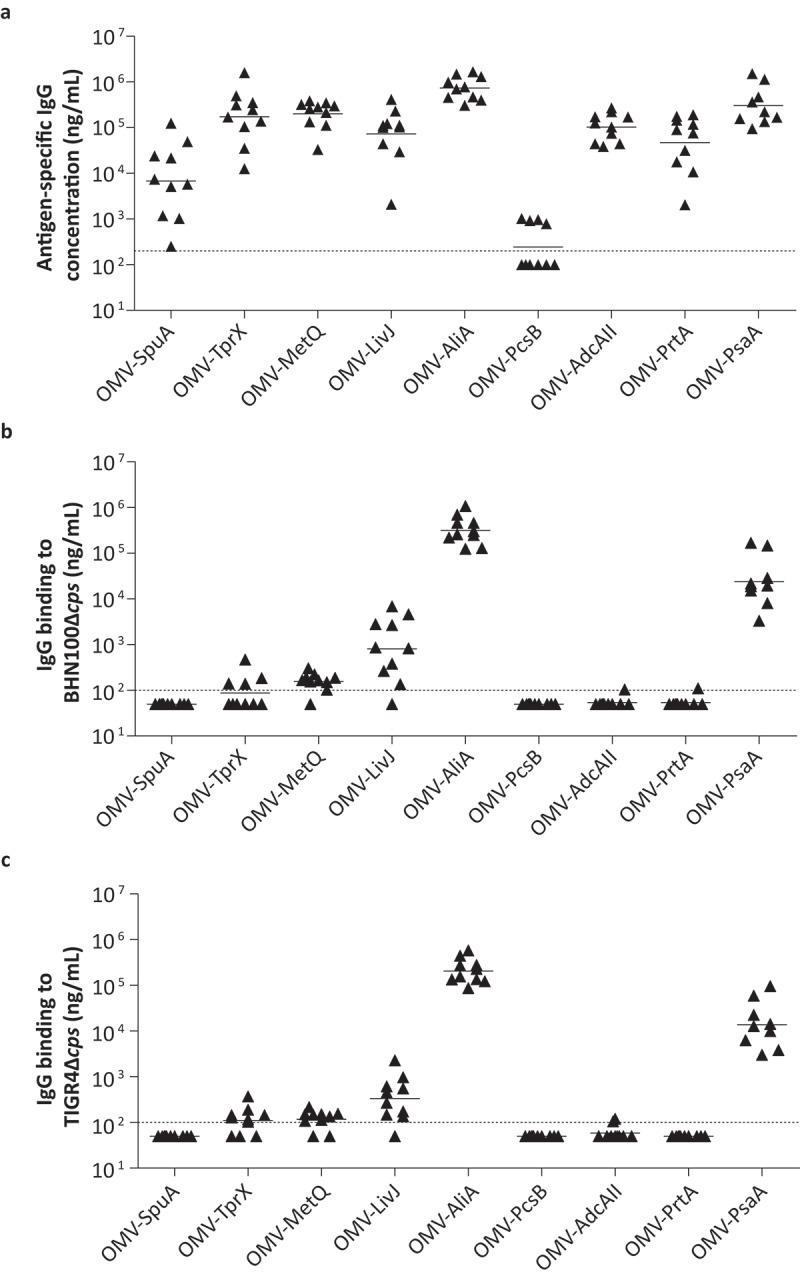
OMVs displaying pneumococcal antigens are highly immunogenic in mice and induced serum antibodies that bind to the bacterial surface. Mice were intranasally vaccinated three times with single antigens coupled to OMVs. n = 10 mice per group, except for OMV-PsaA (n = 9). Sera for IgG measurements were collected from all mice two weeks after the third immunization and analyzed using ELISA. (a) Antigen-specific IgG levels in serum. (b) Binding of IgG in serum to BHN100 capsule mutant (Δ*cps*). (c) Binding of IgG in serum to TIGR4Δ*cps*. Dashed lines indicate detection limit, symbols represent individual mice and horizontal lines indicate the geometric mean of each group.

The OMV-based vaccine formulations were used to vaccinate mice intranasally three times with a two-week interval. Antigen-specific IgG concentrations in serum, collected 2 weeks following the third vaccination, were determined by ELISA (Figure 4(a)). Except for PcsB, detectable antibody levels were observed for all antigens, confirming that at least eight out of nine proteins were immunogenic.

Next, we assessed whether these serum antibodies are capable of binding to the native protein expressed on the surface of two *S. pneumoniae* strains cultured in IVM-CDM using a whole-cell ELISA (Figure 4(b,c)). Unencapsulated strains were used because pneumococci have been shown to downregulate their capsule during colonization to allow nutrient uptake and adhesion to the mucosal surface [35,36]. However, since this process is difficult to be controlled and quantified *in vitro* we have used unencapsulated strains allowing us to study antibody binding in a controlled manner. The ELISA results show that antibodies recognizing AliA, and to a lesser extent PsaA and LivJ bind to the bacterial surface, whereas for all other antigens the antibody binding was close to or below the detection limit.

### Assessment of protection of the selected antigens in a mouse colonization model

**Figure 5.**
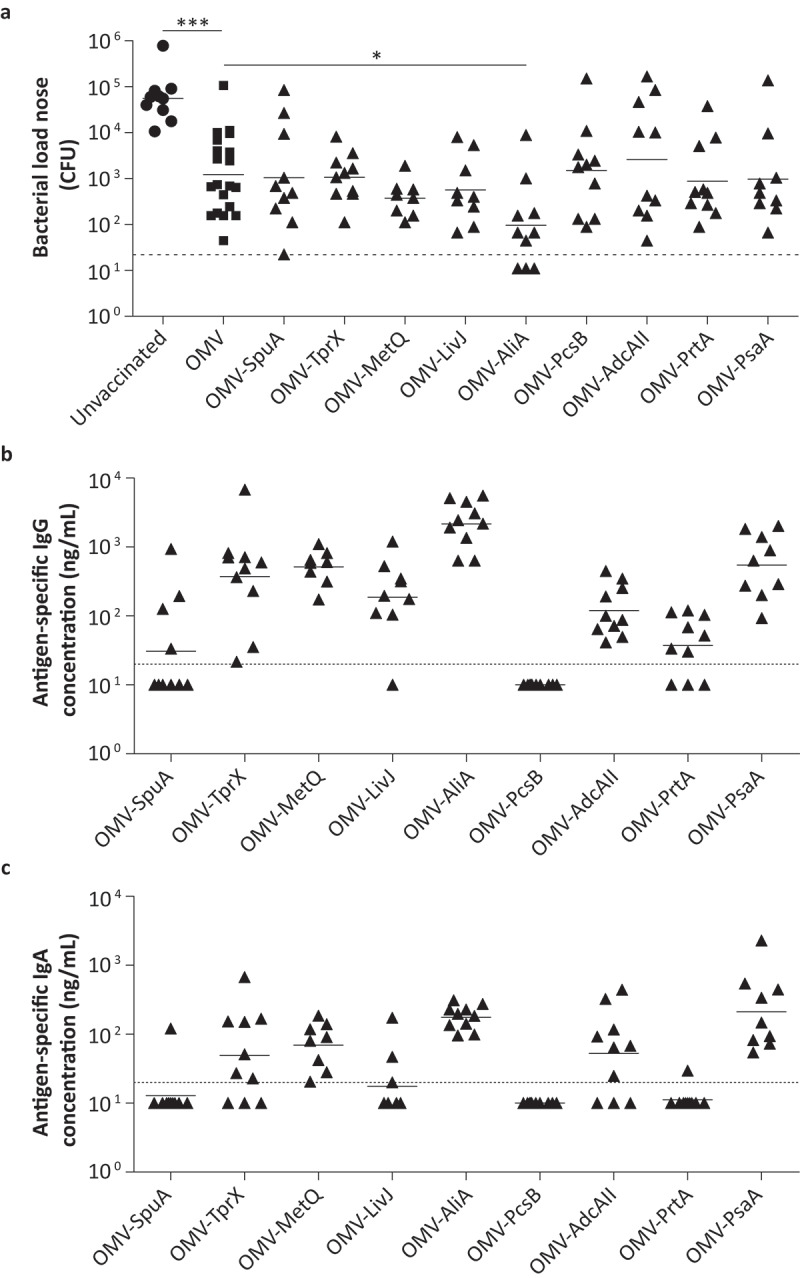
Mucosal protection and antibody levels induced by OMVs displaying selected antigens in a mouse pneumococcal colonization model. Mice were intranasally vaccinated three times with single antigens coupled to OMVs. n = 10 mice per group, except for OMV-PsaA (n = 9) and the OMV control group (n = 20). Three weeks after the last vaccination, mice were infected intranasally with 1 × 10^6^ CFU *S. pneumoniae*. In the OMV-MetQ and OMV-LivJ group 2 and 1 animal(s), respectively, were excluded because of the development of pneumococcal disease. Nasal tissue was harvested 3 d post-infection for further analysis. (a) Pneumococcal load in the nose of mice. (b) Antigen-specific IgG levels in the murine nose by ELISA. (c) Antigen-specific IgA levels in the murine nose by ELISA. Dashed lines indicate detection limit, symbols represent individual mice and horizontal lines indicate the geometric mean of each group. Log_10_-transformed data was used to compare the bacterial load of unvaccinated to OMV vaccinated mice by a two-sided T-test and OMV-antigen vaccinated mice to OMV vaccinated mice by a One-Way ANOVA with Dunnett Post-Hoc testing and 95% confidence intervals. * p-value < 0.05, *** p-value < 0.001.

We subsequently evaluated the capacity of the selected antigens to clear colonization by *S. pneumoniae*. Three weeks after the third vaccination, mice were intranasally infected with *S. pneumoniae* (1 × 10^6^ CFU PBCN0231 in 5 µl) and the nasal bacterial load was determined 3 d later (Figure 5(a)). Mice vaccinated with OMVs displaying no pneumococcal antigen already showed a significant reduction (~70-fold; p-value < 0.0001) in nasal bacterial load compared to unvaccinated mice. Of the tested antigens, only OMVs displaying AliA resulted in statistically significant pneumococcal clearance compared to the OMV control group (p-value = 0.018). These results suggest that, of the nine selected antigens, AliA has the highest protective capacity when coupled to OMVs.

### Immunization with OMVs displaying potential antigens induces mucosal antigen-specific IgG and IgA

To evaluate the local antibody response towards each of the antigens, nasal tissue harvested post-infection was used to measure the mucosal antigen-specific IgG and IgA levels by ELISA. Like the serum antigen-specific IgG levels, the highest median local IgG and IgA levels and the smallest intra-group variation was obtained for antibodies binding to AliA (Figure 5(b,c)). Nasal IgG levels strongly correlated with serum IgG levels (R^2^ = 0.7660; p-value < 0.0001), although nasal concentrations were on average a thousand-fold lower (Figure S5(a)). A less strong correlation (R^2^ = 0.4967; p-value < 0.0001) was seen between the nasal IgG and nasal IgA levels (Figure S5(b)).

### Immunization with OMV-AliA leads to protection against pneumococcal pneumonia

**Figure 6.**
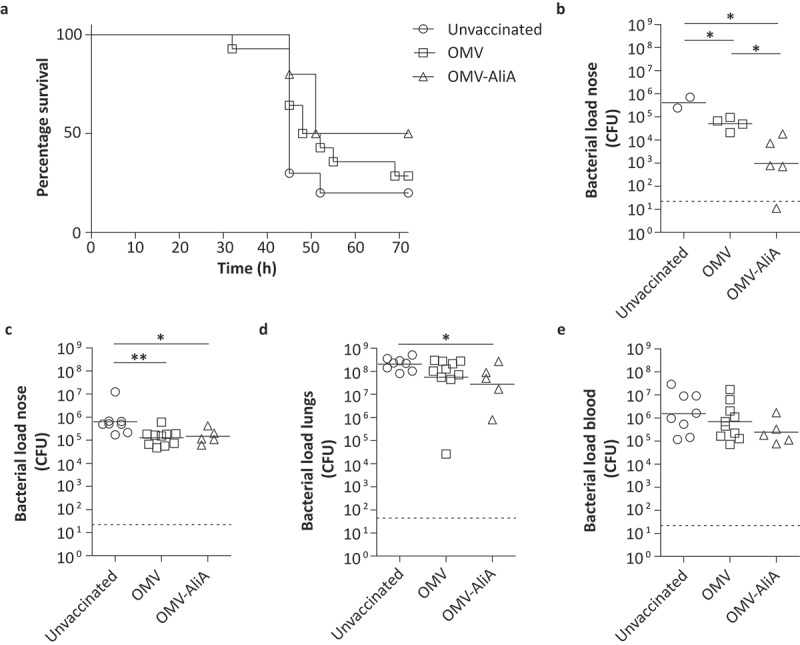
Mice immunized with OMVs displaying AliA show improved survival against pneumococcal disease and a reduced nasal pneumococcal load. Mice were intranasally vaccinated three times with OMVs (n = 14) or OMV-AliA (n = 10) and compared to unvaccinated mice (n = 10). Mice were subsequently infected intranasally with 1 × 10^4.5^ PFU Influenza A followed by 3 × 10^5^ CFU *S. pneumoniae*. Bacterial load was determined in the nose, lungs and blood when mice reached the humane endpoint (clinical signs of invasive disease) or at 3 d post-pneumococcal infection. (a) Survival curve showing when mice reached their humane endpoint and were consequently taken out of the experiment over the course of 72 h. (b) Bacterial load in the nose of mice which survived the 72 h time span without symptoms. (c–e) Bacterial load in the nose (c), lungs (d) and blood (e) of mice which reached their humane endpoint within 72 h. Dashed lines indicate detection limit, symbols represent individual mice and horizontal lines indicate the geometric mean of each group. The survival-like curve was analyzed using Gehan-Breslow-Wilcoxon Test (unvaccinated versus OMV-AliA: p = 0.0590; OMV versus OMV-AliA: p-value = 0.3079; unvaccinated versus OMV: p-value = 0.2751). Statistical significance of the bacterial load was determined using two-tailed T-tests on log_10_-transformed data with 95% confidence intervals. * p-value < 0.05; ** p-value < 0.01.

We then explored whether AliA would also be able to confer protection in a lethal pneumonia co-infection model. A co-infection model was used because a viral infection, in particular an Influenza A infection, of the respiratory tract is associated with increased incidence of pneumococcal disease [37,38]. For this, mice were vaccinated with AliA-loaded OMVs as in the colonization model, resulting in similar serum IgG levels as the mouse colonization model described above (Figure S6(a)). Approximately 3 weeks after the third dose, mice were infected intranasally with influenza virus strain A/Udorn/307/72(H3N2) (1 × 10^4.5^ plaque forming units (PFU) in 10 µl) without inducing signs of disease and in the absence of body weight loss (Figure S6(b)). Three days later, mice were intranasally infected with *S. pneumoniae* (3 × 10^5^ CFU PBCN0231 in 15 µl). Mice were monitored for up to 72 h post-pneumococcal infection for clinical signs of invasive disease and body weight loss (Figure S6(c)). Following 72 h, or in case humane endpoints were reached sooner, mice were removed from the experiment resulting in a survival curve (Figure 6(a)). No symptoms and no significant body weight loss were observed for 50% of the mice vaccinated with OMV-AliA at the 72 h time point. In contrast, only 28% of the OMV control group and 20% of the unvaccinated mice remained asymptomatic (unvaccinated *versus* OMV-AliA: p-value = 0.0590; OMV *versus* OMV-AliA: p-value = 0.3079; unvaccinated *versus* OMV: p-value = 0.2751). When mice were removed from the experiment, the bacterial load in the nose, lungs, and blood were determined. Of the mice that remained asymptomatic over the course of 72 h, OMV-AliA vaccinated mice had a significantly lower nasal bacterial load compared to the two negative control groups: unvaccinated and the OMV-vaccinated group (Figure 6(b)). In addition, the low number of pneumococci detected in the lungs and blood of the mice that survived the first 72 h confirms that these mice were able to control the infection (Figure S6(d,e)). Regardless of the intervention, mice that developed invasive pneumococcal disease had a high bacterial load in the nose, lungs, and bloodstream (Figure 6(c–e)). Of note, a small but statistically significant reduction in nasal bacterial load was observed for diseased mice vaccinated with OMVs (with or without AliA) compared to diseased unvaccinated mice (Figure 6(c)). Taken together, these results highlight the potential of AliA as a vaccine antigen, not only in diminishing pneumococcal colonization but possibly also in reducing invasive disease.

## Discussion

In the present study, we explored the availability of transition metals in the primary niche of *S. pneumoniae* (i.e. the URT) and exploited the outcome to facilitate the identification of relevant vaccine antigens. The concentrations of a range of transition metals were measured in the URT of human volunteers, and used to design a nasal-like medium. Both the pneumococcal growth characteristics as well as the bacterial proteome were strongly influenced by the metal concentrations used in the medium. Finally, nine putative vaccine antigens were selected based on the proteomics results. These antigens were formulated as OMV-based vaccines and tested in relevant mouse models to determine the protective efficacy.

To our knowledge, this is the first comprehensive analysis of the most relevant complexed and free transition metals in the human URT. Iron, copper, and zinc were mainly bound to proteins, whereas both free and protein-bound magnesium and calcium were highly abundant (Figure 1(b)). This suggests that the host limits metal availability by binding metals to proteins, which occurs in variable levels for different transition metals, and consequently restricts bacterial growth in the human nose [15,39–42]. Indeed, host transition metal scavengers, such as calprotectin and lactoferrin have been detected in nasal fluid [43]. The total iron, copper, zinc, and manganese levels measured in this study are similar to the levels measured in mouse nasal tissue [16,23]. Previous attempts to determine the calcium and magnesium levels in human nasal fluid yielded concentrations in the same order of magnitude [22,44]. Furthermore, our data confirm that nasal transition metal levels are deviating from other pneumococcal infection sites, such as the bloodstream and lungs [16,23]. The observed difference in total iron levels between the summer and winter season could potentially be a result of differences in microbiome composition and host nutrient sequestration status [45]. Of note, it is currently unknown whether transition metal levels in adult nasal fluid are different from those found in children and elderly. We showed, based on a small sample size, that nasal fluid of pneumococcal carriage positive and carriage negative adult volunteers of the EHPC model contain similar levels of free and complexed transition metals. In contrast, in mice, zinc levels have been shown to increase in the nasopharynx following *S. pneumoniae* infection, whereas manganese and copper levels do not [16]. However, these differences could potentially be explained by differences in the infection models and the analysis of nasal tissue in mice versus nasal fluid in humans.

The *in vivo*-mimicking culture condition designed in the current study enabled us to investigate *S. pneumoniae* in an *in vivo*-relevant environment. A major advantage of the highly defined medium, as used in this study, is the possibility to change single components, which allowed us to change the transition metal composition, while maintaining the concentrations of the other nutrients.

It is important to note that, *in vivo*, the transition metal levels are likely to be influenced by, (i) uptake and usage by bacteria that are part of the URT microbiome, (ii) the release of transition metals from bacterial or host proteins, and (iii) local changes in transition metal concentrations as a consequence of inflammation [42,46]. These conditions are difficult to simulate and the extent to which these factors contribute to bacterial persistence in the URT is not well defined. Therefore, the IVM-CDM used here was based on the total levels of transition metal levels measured in nasal fluid. Additionally, it should be noted that a broad variety of other nutrients and environmental factors could potentially contribute to the phenotypic differences seen between *in vitro-* and *in vivo*-derived bacteria. This includes nitrogen and carbon sources, of which the levels have been shown to deviate between standard culture media and the human nose [44]. Furthermore, by changing the transition metals concentrations in the medium, the availability of anions changes as well, which might have influenced our observations. We speculate, however, that these changes have a negligible effect on our study because of the generally high concentrations of these anions in both CDM and IVM-CDM. In line with this, only one protein involved in anion transport, the voltage-gated chloride channel family protein (SP_1157), was significantly changed in abundance between the different culture conditions.

Surprisingly, IVM-CDM-adapted pneumococci grew faster compared to CDM-adapted pneumococci (Figure 2(a)). A possible explanation for this could be the higher iron levels in IVM-CDM versus CDM, which is a known virulence determinant [21,47]. Moreover, it is plausible that the ratios between transition metals are more optimal in IVM-CDM compared to CDM, consequently improving bacterial growth. For instance, the manganese to zinc ratio has previously been shown to influence cell division [48]. Strikingly, based on the optical density measurements, IVM-CDM-adapted pneumococci do show autolysis, whereas CDM-adapted pneumococci do not. Autolysis, however, has been associated with increased virulence, immune evasion and the exchange of genetic material, suggesting that IVM-CDM-adapted pneumococci could be more virulent [49,50]. The importance of transition metals in the physiology of *S. pneumoniae*, as shown previously, was confirmed by our whole cell proteomics data [13,16]. In accordance with the literature, the PCA analysis in this study showed that different strains, with differences in serotype and genetic background, have unique proteomic profiles [51]. Nevertheless, the two serotypes examined in the current study responded similarly to differences in metal availability. This observation provides interesting opportunities to find universal strategies to fight pneumococcal infections, e.g. by inhibiting transition metal homeostasis [52].

The adjustment of the medium to a more relevant condition concerning transition metals led to the selection of nine relevant candidate vaccine antigens based on their increased abundance in IVM-CDM *versus* CDM, high conservation level and putative surface-exposure. Interestingly, the majority of these proteins are lipoproteins and function as the substrate-binding component of ATP-binding cassette (ABC)-transporter complexes: TprX is predicted to be involved in the uptake of tryptophan, MetQ is part of a methionine transporter, LivJ is involved in the uptake of leucine, isoleucine, and valine, AdcAII is part of a zinc transporter, PsaA is involved in manganese uptake, and AliA is part of an oligopeptide transporter [53–58]. The selected non-lipoproteins are SpuA, an alkaline amylopullulanase involved in glycogen degradation, PcsB, a peptidoglycan hydrolase and, PrtA, a serine protease [59–61]. Together, this underscores that changes in transition metal availability have a broad impact on the physiology of the bacterium and is thus not limited to proteins directly involved in metal homeostasis [62,63]. Notably, each of the discovered proteins has shown to contribute to the pathogenesis of the bacterium in animal models [11,19,20,53–55,57,59–61,64–68]. In line with this, we found that all nine proteins were expressed during colonization of the murine and human URT at variable levels (Table S4). However, differences in sampling methods between human and mouse samples and differences in pneumococcal loads between samples complicates quantitative analysis of the gene expression data. In accordance with the gene expression results, pneumococcal colonization in mice induced a detectable level of antigen-specific antibodies against PsaA, AliA, MetQ, AdcAII, and PrtA. Anti-PsaA antibodies following colonization have been described before, both in humans and in mice [69–71].

Concerning the protective capacity of the nine selected proteins, all but AdcAII have previously been tested as vaccine antigen in mouse models of pneumococcal disease. In these studies, immunization with PcsB and PrtA significantly reduced the bacterial load in the lungs of mice and PcsB, AliA, PrtA, and PsaA immunization resulted in a significantly prolonged survival time in sepsis models [9,11,30,66,72]. On top of that, PsaA and TprX have been shown to significantly reduce pneumococcal colonization level in mice [73,74]. In contrast, various other studies reported no statistically significant protection against pneumococcal colonization and/or disease following immunization with either MetQ, AliA or LivJ [9,54,75].

Here, we evaluated whether these potential antigens could reduce the nasopharyngeal pneumococcal load in mice when presented on OMVs. *Salmonella* OMVs have previously been shown to be a promising mucosal antigen delivery vehicle with intrinsic adjuvant activity [32,33]. Consistently, significant levels of systemic antigen-specific antibodies were measured for all formulations after the third immunization, except for OMV-PcsB. The lack of an anti-PcsB antibody response might be explained by loss of natural conformational epitopes due to the formation of protein aggregates observed during the OMV-antigen conjugation procedure. Besides intrinsic differences in immunogenicity, the differences in the antibody levels measured with ELISA between the antigens could be explained by differences in antigen-load/immunization, accessibility of epitopes when presented on OMVs and/or accessibility of epitopes upon coating to the ELISA plates. Binding to the bacterial surface was highest for anti-AliA, -PsaA, and -LivJ antibodies, which represents a combined effect of protein abundance and accessibility of the epitopes on the bacterial surface, antibody levels in mouse serum, and the capacity of the antibodies to recognize natural protein epitopes. Overall, this may implicate that immunization with formulations containing AliA, PsaA, and/or LivJ may result in efficient opsonization of pneumococci *in vivo*, leading to clearance. However, it should be noted that the capacity of antibodies to bind to the bacterial surface does not necessarily provide insight into the functionality of these antibodies. Future experiments, such as opsonophagocytosis assays, have to be performed to unravel this. Next to serum antibodies, also antigen-specific mucosal IgG and IgA were detected, although it is yet unknown to what extent these antibodies have contributed to bacterial clearance.

Unexpectedly, we observed nonspecific clearance of colonization following immunization with OMVs lacking a pneumococcal antigen (Figure 5(a)). This nonspecific reduction of pneumococcal colonization, as earlier described for Cholera Toxin Subunit B [76], is possibly mediated by the presence of detoxified LPS, lipoproteins, and other Toll-like receptor (TLR) stimuli in the OMVs. In addition, similar mechanisms of nonspecific protection could potentially have reduced the viral load following Influenza A infection, making these mice less susceptible for the development of pneumococcal disease. Of note, the SpyCatcher-SpyTag technology used for antigen ligation is derived from *S. pyogenes*, and the amino acid sequence shows homology to pneumococcal adhesin-like proteins, which could potentially have contributed to *S. pneumoniae* -specific immunity. However, vaccination with OMVs with or without SpyCatcher had a similar impact on the clearance of pneumococci from the mouse nose (data not shown).

We provided evidence that a relatively low amount of AliA displayed on OMVs (~300 ng/dose) establishes a significant reduction in pneumococcal colonization and protection against disease in mice. Interestingly, AliA has been shown to be important for survival *in vivo* and is thought to play a role in bacterial communication by binding oligopeptides derived from other common colonizers. Peptide uptake by pneumococci *via* AliA has shown to influence the behavior of the bacterium *in vitro*, including reduced growth, reduced capsule thickness, and increased biofilm formation [11,57,58,77]. It is, therefore, tempting to speculate that *in vivo* anti-AliA antibodies could hamper the uptake of oligopeptides by AliA and consequently affect pneumococcal persistence in the host. The results of the present study and the significant protection against sepsis as earlier found, strongly support the relevance of AliA as a vaccine antigen [11]. In contrast to previously published data, PsaA and TprX did not significantly reduce colonization [73,74]. Possibly, this is caused by significant differences in vaccine formulations and the mouse model used, i.e., the use of OMVs as delivery platform with intrinsic adjuvant activity, the relatively low dose of pneumococcal antigen, and the intranasal immunization route.

In conclusion, we established for the first time, a comprehensive overview of the transition metal availability in human nasal fluid. Based on these data we designed a nasal fluid-like medium to gain a better understanding of pneumococcal persistence in the host and the identification of proteins that play a role during colonization. This led to the discovery of AliA as a promising antigen, when displayed on OMVs, as demonstrated by the reduction of the pneumococcal load in the murine nose and the protection against pneumonia in mice.

## Materials and methods

### Ethics statement

#### Human studies

Nasal fluid collection from in total 12 healthy adults (6 males, 6 females) in an age range between 22 and 56 y for measurements of transition metal levels was approved by the medical ethical committee of the Radboudumc Nijmegen and written informed consent was obtained from all volunteers. Volunteers were excluded based on the following criteria: symptoms of a recent common cold, recent nose bleedings or frequent contact with young children.

Oropharyngeal swabs and nasal fluid samples were obtained from healthy, nonsmoking, adult volunteers aged from 18 to 49 y participated in an EHPC study and inoculated intranasally with live pneumococcus, serotype 6B, as described previously [31,78]. Pneumococcal colonization was detected by classical microbiology methods and individuals were defined as pneumococcal colonized, if any nasal wash culture following experimental challenge grew *S. pneumoniae* serotype 6B. Oropharyngeal swabs were obtained from four volunteers that became colonized with *S. pneumoniae*, nasal fluid samples were collected from seven volunteers (n = 3 carriage positive volunteers at day 2 and/or day 7 post-inoculation, n = 4 carriage negative volunteers at day 2 and 7 post-inoculation). All study participants gave written informed consent and research was conducted in compliance with all relevant ethical regulations. Ethical approval was given by the North West National Health Service Research Ethics Committee (REC). Ethics Committee reference number of EHPC study in which oropharyngeal swab were collected: 18/NW/0481. Ethics Committee reference number of EHPC study in which Nasosorption filter samples were collected: 19/NW/0586.

#### Mouse studies

Mice vaccination and/or infection experiments were approved by the Radboudumc Committee for Animal Ethics and conducted according to the Dutch legislation. For all animal experiments, female C57Bl/6 J mice of 7 weeks old were obtained from Charles River in France. Upon arrival, mice were randomized over individually ventilated cages. Mice were allocated to control and experimental groups randomly and the investigators were not blinded. Sterile water and food were provided *ad libitum*. Prior to experimentation, the mice had at least 1 week to acclimate.

### Bacterial strains and growth conditions

*S. pneumoniae* strains BHN100 (19 F) and BHN418 (6B) were used for growth experiments and proteomic analysis. With this, we included two different serotypes and two well-characterized strains, of which BHN418 is used in EHPC studies, in our analysis [31]. BHN100Δ*cps* and TIGR4Δ*cps* [4] were used for whole-cell ELISAs. Bacteria were grown in a standard CDM, prepared as previously described [79]. For IVM-CDM, transition metal levels were adjusted using atomic absorption spectrometry (AAS) standards (Merck, Amsterdam, The Netherlands) (Tables 1 and S6). pH of *in vivo*-mimicking metal mixture was adjusted to a pH of 1.9 using 5 M NaOH. Final pH of both standard CDM and IVM-CDM was 6.5. All stock solutions were stored at −20°C, except for 20% D-glucose (4°C). Just before use, stock solutions were mixed, filter sterilized (0.2 µm) and warmed to 37°C. Bacteria were grown to optical density at 620 nm (OD_620_) of 0.3 and directly mixed with glycerol and stored at −80°C. Glycerol stocks were subsequently used for experiments using a start-inoculum of ~5*10^6^ CFU/mL. For whole-cell shotgun proteomics, bacteria were grown to logarithmic phase (OD 0.3, 15 OD units), harvested by centrifugation (20 min, 3220 g, 4°C), washed once with PBS and stored at −80°C. For mouse infection experiments, Pneumococcal Bacteremia Collection Nijmegen (PBCN) isolate 0231 (4), BHN100 and BHN418 were cultured in Todd-Hewitt broth (Gibco, Thermo Fisher Scientific, Carlsbad, CA, USA) supplemented with yeast extract to OD_620_ 0.25 and stored in glycerol at −80°C. All pneumococcal growth experiments were performed statically at 37°C, 5% CO_2_.

For recombinant protein production, *E. coli* BL21(DE3) was grown in Lysogeny Broth (LB; 10 g/L tryptone, 5 g/L yeast extract, 10 g/L NaCl). For OMV production, *S. typhimurium* SL3261 Δ*tolRA* Δ*msbB* [33] was grown in TYMC (10 g/L tryptone, 5 g/L yeast extract, 2 mM MgSO_4_, 2 mM CaCl_2_). The growth medium was supplemented with 0.2% of glucose. Where appropriate, antibiotics were added to the following concentrations: kanamycin, 50 µg/mL (LB) or 25 µg/mL (TYMC); chloramphenicol 30, µg/mL. Unless stated otherwise *E. coli* BL21(DE3) cultures were incubated at 37°C with shaking and *S*. Typhimurium SL3261 Δ*tolRA* Δ*msbB* at 30°C with shaking.

### Collection of nasal fluid

Human nasal fluid was collected using a Schirmer strip (Daxtrio, Zaandam, The Netherlands), a Nasosorption™ device (Hunt Developments. Midhurst, United Kingdom) or nasal swab UTM™ kit (Copan Diagnostics, Murrieta, California). Nasosorption filters were used in samples obtained from an EHPC study, nasal swabs for sampling the nasopharynx and Schirmer strips for all other measurements. Schirmer strips and Nasosorption devices were incubated for 1 min in the inferior turbinate of the volunteer. Nasal fluid was removed from the collection device by centrifugation (10,000 g, 5 min, 4°C). Nasal fluid was taken (without mucus/cell pellet) and subsequently diluted in H_2_O. To discriminate between protein-bound and free transition metals, diluted nasal fluid was divided into a protein-rich and protein-depleted fraction using Amicon 3 kDa cutoff spin columns (Merck) and centrifugation (40 min, 10,000 g, 4°C).

### Determination of transition metal levels

To determine the transition metal concentrations, nasal samples and culture media were acidified by adding 1% final volume 65% HNO^3-^ in a final volume of 3 mL. Analyses were performed using a model X series I ICP-MS (Thermo Fisher Scientific) or on the iCAP-RQ ICP-MS (Thermo Fisher Scientific) and Cu^2+^, Co^2+^, Zn^2+^, Fe^2+^, Mg^2+^, Mn^2+^ and Ca^2+^ levels were measured in all samples. ICP-MS calibration was performed using ICP multi-element standard solution IV (Merck) ranging from 1 to 100 ppb. Quality control was run using 100 ppb ICP multi-element standard VIII (Merck) and 500 ppb Scandium was used as internal standard.

### Proteomic analysis

#### Sample preparation for nanoLC-MS/MS

Cell pellets were resuspended in PBS, heat-killed (30 min, 56°C) and resuspended in 2.0–2.5 mL 8 M Urea (Merck-Millipore, Darmstadt, Germany) and 2 M thiourea (Sigma-Aldrich/Merck-Millipore) in HPLC-grade water (J.T. Baker/Thermo Fisher Scientific, Center Valley, PA, USA) and disrupted using a microfluidizer (LV1 Low volume Microfluidizer Homogenizer, Microfluidics) at 1000 psi. Lysate was centrifuged (30 min, 3220 g, 4°C) and the supernatant containing solubilized proteins were collected, snap-frozen, and stored at −80°C. Protein concentration was determined with a VarioSkan Flash photometer (Thermo Fisher Scientific) using a Bradford protein assay (Biorad, Munich, Germany) with bovine serum albumin (Sigma-Aldrich/Merck-Millipore) to establish a calibration curve. Five microgram protein of each sample were diluted in 20 mM ammonium bicarbonate (Sigma-Aldrich/Merck-Millipore) solution (ABC) in HPLC grade water (J.T. Baker/Thermo Fisher Scientific) to 50 µl. Trypsin (Promega, Mannheim, Germany) in 20 mM ABC was added in a protein: trypsin ratio of 25:1 and samples were incubated for 17 h at 37°C. The trypic digestion was stopped with acetic acid (Carl Roth GmbH & Co. KG, Karlsruhe, Germany) at a final concentration of 1% (v/v). Subsequently, peptides were purified using C_18_ ZipTip® material (Mettler Toledo, Gießen, Germany) according to the manufacturer’s instructions. After solvent removal by lyophilization, peptides were resuspended in 20 µl LC-buffer [2% (v/v) acetonitrile (ACN; Thermo Fisher Scientific) and 0.1% (v/v) acetic acid (Carl Roth GmbH & Co. KG) in HPLC-grade water (J.T. Baker/Thermo Fisher Scientific)].

#### Data acquisition and analysis

Peptides were separated on an Accucore 150-C_18_ analytical column of 250 mm (25 cm × 75 μm, 2.6 μm C18 particles, 150 Å pore size, Thermo Fischer Scientific) using a Dionex Ultimate 3000 nano-LC system (Thermo Fischer Scientific) and a binary gradient of buffer A [0.1% (v/v) acetic acid (Carl Roth GmbH & Co. KG) in HPLC-grade water (J.T. Baker/Thermo Fischer Scientific)] and buffer B [0.1% (v/v) acetic acid (Carl Roth GmbH & Co. KG) in ACN (J.T. Baker/Thermo Fischer Scientific)] at a flow rate of 300 nl/min. After ionization, peptides were analyzed on a Q Exactive^TM^ (Thermo Fisher Scientific) mass spectrometer in data dependent acquisition (DDA) mode. Further details on the mass spectrometry data acquisition are provided in Table S7.

Raw mass spectrometry data were analyzed with the Proteome Discoverer 2.2 (Thermo Fisher Scientific). Since the annotations of BHN100 and BHN418 database did not match with each other, we used the well-annotated TIGR4 database to analyze all samples at once with protein FDR of <1%. Thus, spectra were searched against a UniProt database limited to *S. pneumoniae* TIGR4 entries (release 03/2017, 2072 entries, www.uniprot.org) supplemented with 245 common laboratory contaminants. During the search, trypsin was set as protease with a maximum of two missed cleavages allowed. Oxidation at methionine was set as variable modification and a maximum false discovery rate of <1% was allowed. Label-free quantification of protein intensities was performed using Proteome Discoverer as well. Raw protein intensities were median normalized over all biological samples. Average intensities per protein were calculated for each strain and medium combination. Ratios were calculated from these average intensities on protein level for each strain to compare the impact of the growth medium. For statistical analysis, a t-test and multiple testing correction according to Benjamini-Hochberg [80] were performed. Proteins displaying a fold change >|1.5| and a q-value < 0.05 were considered as significantly regulated. Proteins that were absent in one condition and present in at least two of three replicates of the other condition were referred to as “OFF”/”ON” proteins, respectively. For PCA a R-based Shiny App was used, only proteins that were identified in each sample were considered and log-transformed protein intensities were used. Localization prediction of proteins was accomplished using PSORTb 3.0 [27]. Volcano plots were likewise created using a Shiny App taking into account multiple testing adjusted q-values (Benjamini-Hochberg) and log_2_-ratios for displaying data. The mass spectrometry proteomics data generated during this study have been deposited to the ProteomeXchange Consortium via the PRIDE [81] partner repository with the dataset identifier PXD014330 and are, in addition, provided in Table S1. The level of amino acid conservation for putative vaccine antigens was assessed by checking the sequence identity using NCBI-BLAST [28]. To this end, the TIGR4 amino acid sequence was blasted against *S. pneumoniae* (taxid:1313) which includes 340 different strains. The maximum number of target sequences was set at 100.

### Production of OMV formulations

#### Construction of plasmids for expression of SpyTag – pneumococcal antigen fusion proteins

pET28 SpT2 MCS was constructed using general cloning techniques to introduce the following DNA sequence between the lac operator and the T7 terminator region of pET28a (the XbaI and XhoI restriction sites are underlined for reference):

5ʹ- tctagaaataattttgtttaactttaagaaggagatataccatgggcagcagccatcatcatcatcatcacagcagcggc

ctggtgccgcgcggcagccatatgggagtgcctactatcgtgatggtggacgcctacaagcgttacaagggtagtggtgg

taccggatccgaattcgagctccgtcgacaagcttgcggccgcactcgagcaccaccaccaccaccactgagatccggct

gctaacaaagcccgaaaggaagctgagttggctgctgccaccgctgagcaataa-3ʹ. Using the KpnI and HindIII restriction sites synthetic DNA encoding the streptococcal antigen sequences were cloned into pET28 SpT2 MCS (sequences shown Table S8), resulting in plasmids pET28 SpT2-SpuA, pET28 SpT2-SP1069, pET28 SpT2-MetQ, pET28 SpT2-LivJ, pET28 SpT2-AliA, pET28 SpT2-PcsB, pET28 SpT2-AdcAII, pET28 SpT2-PrtA and pET28 SpT2-PsaA.

#### Protein purification

*E. coli* BL21 (DE3) cells harboring a pET28 SpT2-antigen fusion expression plasmid were grown in LB containing 0.2% glucose and kanamycin to an OD_600_ of 0.4–0.5. Protein expression was induced by the addition of isopropyl β-D-thiogalactopyranoside (IPTG) to a final concentration of 0.5 mM and the cells were incubated for another two and a half hours. Subsequently, the cells were washed with PBS (pH 7.4) and stored at −20°C. The cells were resuspended in buffer A (50 mM NaPO_4_, 300 mM NaCl, pH 7.4) and phenylmethylsulfonyl fluoride (PMSF) was added to a concentration of 1 mM. The cells were disrupted by two passages through a One Shot cell disruptor (Constant Systems Ltd., Daventry, United Kingdom) at 1.2 kbar. Cell debris and membranes were removed by centrifugation at 10,000 g and 293,000 g, respectively, at 4°C. His_6_-tagged proteins were isolated from the cleared lysate using TALON Superflow medium (GE Healthcare Life Sciences, Eindhoven, The Netherlands) according to the manufacturer’s instructions. Eluates were dialyzed overnight at 4°C against 500 volumes of PBS (pH 7.4). After dialysis glycerol was added to 10% and aliquots were stored at −80°C.

#### OMV isolation

OMVs were isolated from *S*. Typhimurium SL3261 Δ*tolRA* Δ*msbB* cells harboring the pHbpD(Δd1)-SpyCatcher expression plasmid essentially as described [34]. These cells were grown at 30°C in TYMC containing glucose and kanamycin. Fresh medium containing 50 µM of IPTG was inoculated to an OD_600_ of 0.02 and incubated at 30°C, shaking, for 17 h. Cells were removed by two successive centrifugation steps at 5000 g. The supernatant was passed through 0.45 µm-pore-size membrane filter (Merck-Millipore) and centrifuged at 235,000 g for 75 min to sediment the OMVs. The vesicles were resuspended in PBS (pH 7.4) containing 15% glycerol.

#### Ligation of antigens to Hbp in OMVs

OMVs containing HbpD(Δd1)-SpyCatcher and purified SpT2-antigen fusion protein were mixed and incubated for 24 h at 4°C. After the addition of ~25 volumes of PBS, the OMV suspension was passed through a 0.45 µm-pore-size membrane filter (Merck-Millipore). The OMVs were collected by centrifugation at 208,000 g for 75 min and resuspended in ~6 volumes of PBS containing 500 mM NaCl. Finally, the OMVs were collected by centrifugation at 293,000 g for 60 min and resuspended in PBS containing 15% glycerol to a concentration of 2 OD units of OMVs per µL. One OD equivalent (OD_eq_) unit of OMVs is defined as the amount of OMVs derived from 1 mL of cells with an OD_660_ of 1. Final vaccine formulations were run on a SDS-PAGE gel followed by Coomassie staining to confirm antigen ligation to OMVs.

### Mouse immunization and infection experiments

Mice were intranasally immunized three times under inhalation anesthesia with 5 µL OMVs with a 2 weeks interval. Each dose contained 8 OD units of OMVs displaying (a fragment of) one out of nine selected proteins in PBS with 15% glycerol, except for the second immunization in the pneumococcal disease experiment: OMV and OMV-AliA vaccinated mice received 4 OD units in 5 µL (half of the original dose). Two weeks after the third immunization blood was collected from the tail vein for antibody analysis by ELISA.

For colonization experiments, 3 weeks after the third vaccination, mice were intranasally challenged with 1 × 10^6^ CFU *S. pneumoniae* PBCN0231 in 5 µL PBS under inhalation anesthesia. Three days post-infection, mice were euthanized, and nasal tissue was harvested, homogenized and used for plating on blood agar plates to determine the bacterial load. Lower limit of detection: 22 CFU/animal. Remaining homogenized nasal tissue was snap frozen and stored for antibody measurements by ELISA.

For the pneumococcal disease experiment, 18 d after the third immunization, mice were infected with 10^4.5^ PFU influenza virus strain A/Udorn/307/72(H3N2) in 10 µL PBS without anesthesia. Three days post-influenza infection mice were infected with 3 × 10^5^ CFU *S. pneumoniae* PBCN0231 in 15 µL PBS under inhalation anesthesia. Over the course of 72 hours, mice were scored based on clinical symptoms indicating the development of invasive disease (body weight loss, hunched back, ruffled coat, reduced mobility) and where removed from the experiment just before they reached their humane endpoint. Blood, homogenized nasal tissue and homogenized lung tissue were plated to determine the bacterial load as described above. Lower limit of detection: nasal tissue 22 CFU/animal, lung tissue 44 CFU/animal and blood 22 CFU/animal.

### Protein immunogenicity during colonization

Mice were intranasally challenged with 1 × 10^6^ CFU *S. pneumoniae* BHN100 in 5 µL PBS under inhalation anesthesia. Four weeks post-infection, mice were sacrificed and blood was collected. Serum was used for antigen-specific antibody measurement by ELISA.

### Antibody measurements by ELISA

Maxisorp high binding affinity plates (Thermo Fisher Scientific) were coated with 5 μg/mL purified antigen or heat-killed (30 min, 56°C) BHN100Δ*cps*/TIGR4Δ*cps* OD_620_ 0.3 previously grown in IVM-CDM, in PBS at 4°C overnight. The next day, wells were blocked with 1% BSA (Sigma) and subsequently incubated for 1 h at 37°C with serum or homogenized nasal tissue from individual mice. Thereafter, the wells were incubated with anti-mouse IgG-alkaline phosphatase (Sigma) or anti-mouse IgA-alkine phosphatase (Southern Biotech, Birmingham, Alabama) for 1 h at 37°C. Between incubation steps, all wells were washed with PBS containing 0.05% Tween-20 (Merck). Samples were developed using 1 mg/mL *p*-nitrophenylposphate in substrate buffer (1 M diethanolamine, 0.5 mM MgCl_2_ pH 9.8) and the OD_405_ was measured 20 min after substrate addition.

### *In vivo* gene expression analysis

For the analysis of pneumococcal gene expression in the mouse URT, mice were intranasally challenged with 1 × 10^6^ CFU *S. pneumoniae* BHN418 in 5 µL PBS under inhalation anesthesia. Three days post-infection, mice were sacrificed and bacteria were collected by a nasal wash with 1 mL PBS. A fraction of the nasal wash was used to determine the bacterial load as described above. Remaining sample was directly mixed with RNA Protect Bacterial Reagent (Qiagen, Hilden, Germany) and bacterial pellets were snap frozen and stored at −80°C until further analysis.

Analysis of pneumococcal gene expression in the human URT was performed using samples obtained from the EHPC study conducted in Liverpool in 2018–2019, as previously described [31,78]. Oropharyngeal swabs of carriage positive volunteers were collected in 500 µL of RLT lysis buffer (Qiagen) at several time points and stored directly after collection at −80°C, until RNA extraction.

Total RNA from individual mouse and individual human samples was extracted using RNeasy kit (Qiagen) followed by DNase treatment (DNAfree, Invitrogen, Thermo Fisher Scientific). RNA was amplified using MessageAmp II-Bacteria RNA Amplification Kit (Invitrogen, Thermo Fisher Scientific) and subsequently reverse transcribed using SuperScript One-Cycle cDNA kit (Invitrogen, Thermo Fisher Scientific). qPCR analysis was performed using undiluted cDNA from individual mouse or human samples, Sso SYBR Green advanced mix (Bio-Rad, Hercules, California) and quantitively validated primer sets (Table S9). MilliQ and BHN418 gDNA were included as negative and positive controls, respectively. For mouse samples, only nasal washes with a recovered bacterial load >2 × 10^4^ CFU/mL were included, because the low bacterial load might induce bias in the qPCR analysis. Moreover, in all cases a Cq value < 39.0 and a melting temperature matching with the positive control was required to be included in the final dataset. To exclude the presence of gDNA, qPCR analysis was performed on template material obtained following a cDNA reaction without reverse transcriptase. For none of the samples, this did result in a product with a Cq value < 39.0.

### Statistics

Statistical analyses were all performed using GraphPad Prism version 5.03 (GraphPad Software). Details on the statistical parameters used can be found in the figure legends. Transition metal levels were analyzed using two-tailed Mann-Whitney U tests. For mouse nasal bacterial load in the colonization experiment, a One-Way ANOVA, including a Dunnett Post-Hoc test, was used on log10-transformed data with OMV vaccinated mice as control group. In the pneumococcal disease mouse experiment, the log10-transformed bacterial load was compared by two-tailed T-tests. Survival-like curve analysis was performed using a Gehan-Breslow-Wilcoxon Test and correlations between antibody levels were checked using log10-transformed data and Pearson correlations. P-value < 0.05 was considered significant and p-values are expressed as follows: * p-value < 0.05, ** p-value < 0.01, *** p-value < 0.001.

## Supplementary Material

Supplemental Material

## Data Availability

The mass spectrometry proteomics data generated during this study have been deposited to the ProteomeXchange Consortium via the PRIDE (81) partner repository with the dataset identifier PXD014330 (https://www.ebi.ac.uk/pride/archive?keyword=PDX014330) and are, in addition, provided in Table S1. All other data supporting the findings of this study are available within the article and/or its supplementary materials.
