# Supplementary material for: Exploring metal availability in the natural niche of Streptococcus pneumoniae to discover potential vaccine antigens
Source: Virulence. 2020 Oct 5;11(1):1310–28. doi: 10.1080/21505594.2020.1825908 (PMC7550026; doi:10.1080/21505594.2020.1825908)
Supplement: Supplemental Material [file KVIR_A_1825908_SM6912.zip › captions.docx]

**Fig. S1. Nasal fluid obtained from nasopharynx and inferior turbinate contain similar levels of transition metals.** Nasal fluid was collected from the inferior turbinate and from the nasopharynx from ten adults during the summer season. Mg^2+^, Ca^2+^, Fe^2+^, Cu^2+^, Zn^2+^, Co^2+^, and Mn^2+^ concentrations were determined using ICP-MS. Data from the inferior turbinate corresponds to the data shown in Fig. 1A. The dashed line indicates detection limit, symbols represent individual volunteers and horizontal lines indicate the geometric mean of each group. Co^2+^ and Mn^2+^ levels were below detection limit (<40 µg/L) for all volunteers at both anatomical sites measured. Statistical significance was determined using a two-tailed Mann-Whitney U test with 95% confidence intervals; * p-value<0.01.

**Fig. S2. Transition metal levels in nasal fluid of *S. pneumoniae* carriage positive and carriage negative individuals are similar.** Nasal fluid of seven adult volunteers experimentally inoculated with *S. pnuemoniae* was collected from the inferior turbinate using a Nasosorption device. Samples were collected prior to inoculation (day 0; circles) and 2 days (squares) and 7 days (triangles) post inoculation. Volunteers were classified into carriage negative volunteers (n=4; closed symbols) and carriage positive (n=3; open symbols) by classical microbiology methods. Mg^2+^, Ca^2+^, Fe^2+^, Cu^2+^, Zn^2+^, Co^2+^, and Mn^2+^ concentrations in protein depleted and protein-rich nasal fluid fractions, representing free (A) and protein-bound (B) transition metals, respectively, were determined using ICP-MS. Dashed line indicates the detection limit, symbols represent individual volunteers. Co^2+^ levels were below detection limit (<1.5 µg/L) for all volunteers during all measurements.

**Fig. S3. Antigens discovered under *in vivo*-mimicking conditions are immunogenic during pneumococcal colonization.** Naive mice (n=5) were intranasally infected with 1x10^6^ CFU *S. pneumoniae*. Serum was collected four weeks post-infection and used for the evaluation of protein-specific antibodies by ELISA. Dashed line indicates detection limit, symbols represent individual mice and horizontal lines indicate the geometric mean of each group.

**Fig. S4. Confirmation antigen coupling to OMVs.** Recombinantly produced antigens with SpyTag (SpT) sequence were coupled to *Salmonella* OMVs displaying HbpD-SpyCatcher (HbpD-SpC). Equivalent volumes of vaccine stocks containing equivalent amounts of OMVs, based on OD units, were run on a SDS-PAGE gel followed by Coomassie staining. Coupling of antigens to OMVs was confirmed the difference in electrophoretic mobility of the HbpD-SpC-SpT-Antigen fusion protein compared to the HbpD-SpC protein. The HbpD-SpC-SpT-Antigen fusion protein is indicated with a black arrowhead, possible degradation products are indicated with open arrowheads and asterisks indicate major outer membrane proteins intrinsically present in the OMV membrane**.**

**Fig. S5. Correlation of mucosal and serum antibodies following immunization with OMVs displaying single antigens.** Mice (n=10/group except OMV-PsaA: n=9) were intranasally vaccinated three times with single antigens coupled to OMVs. Antigen-specific IgG levels in serum were measured by ELISA using serum collected two weeks after the third immunization (see also Fig. 4A). Mice were subsequently infected intranasally with 1x10^6^ CFU *S. pneumoniae* and nasal tissue was harvested three days post-infection for nasal IgG and IgA measurements by ELISA. A) Correlation between nasal antigen-specific IgG and serum antigen-specific IgG in all mice, combined for all antigens. B) Correlation between nasal antigen-specific IgG and nasal antigen-specific IgA in all mice, combined for all antigens. Symbols represent individual mice. Statistical analysis was performed on log-transformed data using Pearson correlation.

**Fig. S6. Antigen-specific IgG levels, body weight loss, and pneumococcal load of mice in a lethal pneumonia model.** Mice were intranasally vaccinated three times with OMVs (n=14) or OMV-AliA (n=10) and compared to unvaccinated mice (n=10). Serum for antigen-specific IgG measurement was collected two weeks after the third immunization. Mice were subsequently infected intranasally with 1x10^4.5^ PFU Influenza A followed by 3x10^5^ CFU *S. pneumoniae* three days later. The body weight was measured once a day until mice reached the humane endpoint (clinical signs of invasive disease) or until three days post-pneumococcal infection. A) AliA-specific antibody levels in serum by ELISA. B) Body weight loss (%) of mice post-influenza infection compared to body weight pre-influenza infection with a positive percentage indicating a decrease in body weight. C) Body weight loss (%) of mice post-pneumococcal infection compared to body weight pre-pneumococcal infection with a positive percentage indicating a decrease in body weight. D) Bacterial load in the lungs of mice that remained asymptomatic up to three days post-pneumococcal infection. E) Bacterial load in the bloodstream of mice that remained asymptomatic up to three days post-pneumococcal infection. Dashed line indicates detection limit, symbols represent individual mice and horizontal line indicates the geometric mean.
