# Supplementary material for: Exploring metal availability in the natural niche of Streptococcus pneumoniae to discover potential vaccine antigens
Source: Virulence. 2020 Oct 5;11(1):1310–28. doi: 10.1080/21505594.2020.1825908 (PMC7550026; doi:10.1080/21505594.2020.1825908)
Supplement: Supplemental Material [file KVIR_A_1825908_SM6912.zip › Table_S5.docx]

**Table S5.** **Antigen load per OMV dose (8 OD_eq_) based on quantitative densitometry on SDS-PAGE of OMV-formulations**

| Formulation | Antigen per dosis OMV (ng) | Outer membrane proteins (40, 37, 35, 28 kDa) per dosis (µg) |
| --- | --- | --- |
| OMV-HbpD-SpC | - | 7.8-10.4 |
| OMV-SpuA | 97 | 7.0 |
| OMV-TprX | 180 | 7.7 |
| OMV-MetQ | 203 | 7.1 |
| OMV-LivJ | 273 | 8.2 |
| OMV-AliA | 311 | 5.8 |
| OMV-AdcAII | 315 | 11.7 |
| OMV-PrtA | 11 | 10.2 |
| OMV-PsaA | 263 | 10.6 |
| OMV-PcsB | 65 | 10.0 |
