# Supplementary material for: Exploring metal availability in the natural niche of Streptococcus pneumoniae to discover potential vaccine antigens
Source: Virulence. 2020 Oct 5;11(1):1310–28. doi: 10.1080/21505594.2020.1825908 (PMC7550026; doi:10.1080/21505594.2020.1825908)
Supplement: Supplemental Material [file KVIR_A_1825908_SM6912.zip › Table_S7.docx]

**Table S7. Nano LC-MS/MS data acquisition parameters.**

| **LC-Parameters** | |
| --- | --- |
| Instrument | Ultimate 3000 RSLC (Thermo Fisher Scientific) |
| Trap column | Acclaim PepMap, 2 cm, 100 µm, 100 Å pore size (Thermo Fisher Scientific) |
| Analytical column | Accucore, 25 cm, 2.6 µm, 150 Å pore size (Thermo Fisher Scientific) |
| Buffer system | Binary buffer system consisting of 0.1% acetic acid in *A. dest.* (buffer A) and 0.1% acetic acid in 100% ACN (buffer B) |
| Flow rate | 300 nl/min |
| Gradient | 0 min 2% B 🡪  2 min 5% B 🡪  10 min 5% B 🡪  130 min 25% B 🡪  135 min 40% B 🡪  137 min 90% B 🡪  142 min 90% B 🡪  145 min 2% B 🡪  155 min 2% B |
| Gradient length | 155 min |
| Column oven temperature | 40°C |
| **MS-Parameters** | |
| Instrument | Q Exactive mass spectrometer (Thermo Fisher Scientific) |
| Operation mode | Data-dependent acquisition |
| **Full MS-Parameters** | |
| MS scan resolution | 70,000 |
| AGC target | 3e6 |
| Maximum ion injection time for the MS scan | 120 ms |
| Scan range | 350 to 1650 m/z |
| Spectra data type | Centroid |
| **MS2-Parameters** | |
| Resolution | 17,500 |
| MS/MS AGC target | 2e5 |
| Maximum ion injection time for the MS/MS scans | 120 ms |
| Selection for MS/MS | 10 most abundant isotope patterns with charge ≥2 from the survey scan |
| Isolation window | 3 m/z |
| Fixed first mass | 100 m/z |
| Dissociation mode | Higher energy collisional dissociation (HCD) |
| Normalized collision energy | 27.5% |
| Dynamic exclusion | 30 s |
| Charge exclusion | 1, >7 |
