# Supplementary material for: Exploring metal availability in the natural niche of Streptococcus pneumoniae to discover potential vaccine antigens
Source: Virulence. 2020 Oct 5;11(1):1310–28. doi: 10.1080/21505594.2020.1825908 (PMC7550026; doi:10.1080/21505594.2020.1825908)
Supplement: Supplemental Material [file KVIR_A_1825908_SM6912.zip › Table_S8.docx]

**Table S8. DNA and amino acid sequences of antigens used**

| **Antigen (locus)** | **DNA sequence (5’-3’, from the kpni restriction site to the hindiii restriction site) derived from *S. pnuemoniae* TIGR4** | **Amino acid sequence of the encoded antigen fragment** |
| --- | --- | --- |
| **SpuA (SP_0268)** | ggtaccggaagcgatgaaagtacgccaactacgaatgagccaaacaatcgtaacactacgactttagcccagcccttgacggatactgcggcaggcagtgggaaaaacgaatctgacatctcctcaccagggaacgccaacgcttcattagagaagacggaagaaaaacctgcgacggaaccaacgactccagcggcctcaccagccgatcctgctccacaaacgggccaagaccgcagtagtgagcccacgacctctacttcgccagtaacaaccgaaacgaaagcagaggagccaatcgaggacaactatttccgtattcacgtaaaaaaattgccggaggagaacaaggatgcacagggactgtggacttgggatgacgttgagaagccatccgagaactggcccaacggtgccttatcattcaaagatgccaaaaaagacgattacgggtattatctggatgtaaagttgaaaggagaacaagctaaaaagatttctttcctgattaataacaccgccggcaagaatttgacgggcgataaatccgtagaaaaattggtgcccaagatgaatgaagcatggttggatcaggactacaaagtgttcagttatgaaccccaaccggcagggaccgttcgtgttaactattaccgcaccgacgggaactatgacaaaaaatcactgtggtactggggggacgtaaaaaacccttctagtgcccaatggcctgacggtacggattttacagcaaccgggaagtatggtcgctatatcgacattccacttaatgaggcggcgcgtgaatttggattcctgttgttggacgaatccaaacagggggatgatgtgaagattcgtaaagaaaattataaatttacagaccttaagaatcattctcagatttttcttaaagacgatgatgagagcatttatacgaatccgtattatgtgcacgacattcgtatgacgggcgcacagcatgttgggaccagcagtattgaaagcagcttttccaccttagttggagcgaaaaaggaggacatcctgaagcatagcaatattacgaaccacttgggaaacaaggtaacaattacagacgtagcgattgatgaagctggaaaaaaggtgacctatagtggggacttctcggataccaagcacccctacacagtttcttataatagcgatcaatttacgaccaagacctcatggcgtctgaaggatgagacctatagttatgacggaaaacttggggctgatcttaaagaggaaggaaaacaagtagacttaactctttggtcaccgtctgccgataaggtatcagtcgttgtctacgacaagaatgatcccgacaaggttgttggtactgtggcgttagaaaaaggggagcgcggaacctggaagcaaacattagacagcaccaataaacttggtatcacagactttacggggtactactaccagtaccaaatcgaacgtcaaggaaagaccgtgcttgccctctgaaagctt | SDESTPTTNEPNNRNTTTLAQPLTDTAAGSGKNESDISSPGNANASLEKTEEKPATEPTT  PAASPADPAPQTGQDRSSEPTTSTSPVTTETKAEEPIEDNYFRIHVKKLPEENKDAQGLW  TWDDVEKPSENWPNGALSFKDAKKDDYGYYLDVKLKGEQAKKISFLINNTAGKNLTGDKS  VEKLVPKMNEAWLDQDYKVFSYEPQPAGTVRVNYYRTDGNYDKKSLWYWGDVKNPSSAQWPDGTDFTATGKYGRYIDIPLNEAAREFGFLLLDESKQGDDVKIRKENYKFTDLKNHSQIF  LKDDDESIYTNPYYVHDIRMTGAQHVGTSSIESSFSTLVGAKKEDILKHSNITNHLGNKVTITDVAIDEAGKKVTYSGDFSDTKHPYTVSYNSDQFTTKTSWRLKDETYSYDGKLGADLKEEGKQVDLTLWSPSADKVSVVVYDKNDPDKVVGTVALEKGERGTWKQTLDSTNKLGITDFTGYYYQYQIERQGKTVLAL |
| **TprX (SP_1069)** | ggtaccggtcagaataacaaagatgagaaaaagattacaaagatcggagtgttacaattcgtttcgcacccatccttggatttgatttacaagggcatccaagacggattggccgaagagggatacaaagacgaccaggtaaaaattgattttatgaatagtgaaggcgaccaaagtaaggtcgccaccatgtcaaaacagcttgtggcgaatgggaacgatttagtagtaggcatcgctacaccggcagcacagggacttgcgtccgcaacaaaggacctgcccgtgattatggccgcaatcacagatccaatcggggctaatctggtgaaagacttgaagaaacctgggggaaatgtaaccggcgtgagtgaccacaacccggcgcagcagcaggtcgaactgattaaagctcttacgcccaacgtaaagacgattggtgctctgtattcgagttccgaggataattctaagacgcaagtcgaggaatttaaggcttacgccgagaaagccggccttaccgtggaaacctttgcggtgccatcgacaaatgagatcgccagcacagtcaccgtgatgacctctaaagtagacgctatttgggtgccgattgataacacaatcgcatcaggttttcctaccgtggtctcttcaaaccaaagttctaaaaaaccgatctacccatcagctaccgcgatggtagaggtcggcgggttggcttccgttgtcattgatcaacatgatcttggcgttgccacaggcaagatgatcgtacaggttttgaaaggagcaaaaccggcggatacccctgtcaatgtcttttccaccggcaagagcgttattaacaagaaaattgcacaagaactgggtattaccatccctgaatctgtactgaaagaagcagggcaggttatcgaatgaaagctt | QNNKDEKKITKIGVLQFVSHPSLDLIYKGIQDGLAEEGYKDDQVKIDFMNSEGDQSKVAT  MSKQLVANGNDLVVGIATPAAQGLASATKDLPVIMAAITDPIGANLVKDLKKPGGNVTGVSDHNPAQQQVELIKALTPNVKTIGALYSSSEDNSKTQVEEFKAYAEKAGLTVETFAVPST  NEIASTVTVMTSKVDAIWVPIDNTIASGFPTVVSSNQSSKKPIYPSATAMVEVGGLASVVIDQHDLGVATGKMIVQVLKGAKPADTPVNVFSTGKSVINKKIAQELGITIPESVLKEAGQVIE |
| **MetQ (SP_0149)** | ggtaccggtgacaatgccaccacgattaaaattgctaccgtcaaccgctccggatctgaggaaaaacgctgggataaaatccaagaacttgtcaaaaaagacggaattacgttagaatttacagagttcacagactactctcaacccaacaaagcgacggcggatggggaagtggacttaaatgcgtttcagcactacaatttccttaacaactggaataaagagaatggaaaggatcttgttgctattgctgacacttatatttcgcccatccgcttgtactcaggattaaatggtagcgctaacaagtacacaaaggtcgaggatattcctgcaaatggcgaaattgcggttcctaatgacgccacgaacgaatcgcgtgctctttatctgcttcagtcggctggccttattaaactggatgtgagcggaacggcgttggcaacggttgcaaatattaaggaaaatcccaagaacttgaagatcacagaattggacgcctcacagaccgcgcgttcattaagtagtgttgacgcagccgtagtgaacaatacgtttgtcactgaggccaaattagactacaaaaagtcgcttttcaaggagcaggccgatgaaaacagtaagcaatggtataacattattgtcgctaaaaaagactgggagactagccccaaggctgacgctattaaaaaagttattgcagcataccacacagatgatgtcaagaaggtgattgaagaatcctctgatggtctggatcagccggtatggtgaaagctt | DNATTIKIATVNRSGSEEKRWDKIQELVKKDGITLEFTEFTDYSQPNKATADGEVDLNAF  QHYNFLNNWNKENGKDLVAIADTYISPIRLYSGLNGSANKYTKVEDIPANGEIAVPNDAT  NESRALYLLQSAGLIKLDVSGTALATVANIKENPKNLKITELDASQTARSLSSVDAAVVN  NTFVTEAKLDYKKSLFKEQADENSKQWYNIIVAKKDWETSPKADAIKKVIAAYHTDDVKK  VIEESSDGLDQPVW |
| **LivJ (SP_0749)** | ggtaccggtgtcaacacagcggggaactctgtggaagaaaagactattaaaattggtttcaatttcgaggagtcgggctccttagcggcgtacggtactgctgagcagaaaggagcccaacttgccgtcgatgagatcaacgcagctgggggcattgacggaaagcaaatcgaagtagtagataaggacaacaaaagtgagactgctgaagctgcaagcgtcaccaccaatctggtgacacaatcaaaggtttccgcggtcgtgggccctgcaacgtctggtgctaccgcagccgccgttgctaatgcaactaaggcaggtgttccattgatctcaccgtccgccactcaggatggtctgaccaagggtcaagactaccttttcatcgggacgtttcaagactccttccaaggcaaaattatctcgaattatgtcagcgaaaagctgaacgctaaaaaagttgttttatataccgacaacgcatccgattacgcgaaaggaattgcgaagtccttccgcgagtcatacaagggcgagatcgttgcggatgagacctttgttgcaggtgacacggactttcaagcagctcttacaaaaatgaagggaaaggactttgacgcgattgtagtccctggttattataatgaggcagggaagatcgtaaatcaggcacgcggtatgggcattgacaagccgattgttggcggggatggatttaacggcgaagagttcgtgcaacaggcgacagcggaaaaagcctcaaacatctactttattagcggattctccacgactgttgaggtaagcgcgaaagctaaggcgttccttgatgcgtatcgcgctaagtataacgaagagccatcgacattcgcggccttagcgtacgattcggttcatttggtggcaaacgctgctaaaggtgcgaagaactcgggtgagattaaagacaatttggcgaagactaaagactttgagggggtcactggtcagactagctttgacgcggatcacaacacggtaaagactgcatatatgatgactatgaataatggaaaggtggaagcggctgaggtcgtaaaaccatgaaagctt | VNTAGNSVEEKTIKIGFNFEESGSLAAYGTAEQKGAQLAVDEINAAGGIDGKQIEVVDKD  NKSETAEAASVTTNLVTQSKVSAVVGPATSGATAAAVANATKAGVPLISPSATQDGLTKG  QDYLFIGTFQDSFQGKIISNYVSEKLNAKKVVLYTDNASDYAKGIAKSFRESYKGEIVAD  ETFVAGDTDFQAALTKMKGKDFDAIVVPGYYNEAGKIVNQARGMGIDKPIVGGDGFNGEEFVQQATAEKASNIYFISGFSTTVEVSAKAKAFLDAYRAKYNEEPSTFAALAYDSVHLVAN  AAKGAKNSGEIKDNLAKTKDFEGVTGQTSFDADHNTVKTAYMMTMNNGKVEAAEVVKP |
| **AliA (SP_0366)** | ggtaccggttctggctccggctccagcacaaaaggggaaaagaccttctcatatatttacgaaaccgatccagataacctgaattaccttactactgctaaggctgcaacggctaatattacgtctaacgtggtcgatggattgttagaaaatgatcgctacggaaactttgtgccgtctatggccgaagattggtccgtatctaaggatggtttgacttacacgtacaccattcgtaaagacgccaaatggtacacctcagaaggcgaggaatatgcagcagtcaaagcccaggacttcgttacagggctgaagtacgcagccgacaaaaaaagcgatgctctgtacctggtacaggaatcaatcaagggcctggatgcttacgtgaaaggcgaaatcaaagacttcagtcaggtcggcattaaggccctggatgaacaaactgtccagtacacgttgaataaaccagaatctttttggaatagcaaaacgacaatgggtgtgctggcgcccgtaaacgaagagtttttaaattctaagggcgatgattttgccaaggccaccgaccctagttctctgctgtacaatggaccgtatttattgaagtctatcgtgaccaaatcgtccgtagaatttgcgaaaaaccctaattattgggataaggataacgtccacgtggacaaagtaaaacttagcttttgggatggccaagacacatcgaagccggcggaaaactttaaggacgggtcgttgacggccgcgcgtttatacccgacatccgcatcctttgctgagctggaaaagagcatgaaggataacatcgtttatacgcaacaggactcaatcacgtacttagttgggacgaatattgatcgccaatcctacaagtacacgagcaaaacaagcgacgagcagaaagcgagtacaaaaaaggctttgttaaataaggacttccgccaagccatcgcgtttggatttgaccgcaccgcctatgcgtcccagttgaacggacagaccggggcgtcgaagattcttcgcaatctgtttgtccctcccacatttgtacaggctgacggcaaaaatttcggagatatggtaaaagaaaagttagtgacctacggtgacgaatggaaagatgtgaacttggcggattcccaagatggtttgtataacccggaaaaggccaaagcagaatttgctaaagcaaaatccgcattacaagcagagggggtgcaatttccgatccaccttgatatgcccgtagaccaaacggctaccacaaaggtgcagcgcgtgcaatcaatgaagcagagccttgaggctactttaggagcggacaatgtgattattgacatccaacaactgcaaaaagacgaagttaacaatattacatattttgcggagaacgcggccggggaagactgggatctgtccgataacgtggggtggggaccagactttgcggacccgagcacttacttagatattattaagcctagcgtcggcgagagcacaaagacctacttggggtttgatagtggggaagataacgtcgcggcaaagaaggtcggcttatacgactacgaaaagctggttactgaagcaggcgacgagacaacagacgttgcaaaacgttacgataaatatgctgctgcccaagcatggcttactgactctgcgcttattatcccaactacctcgcgcacgggccgtccaatcctgtccaaaatggtgcctttcactatcccatttgcgttgtccggcaataagggaacctctgagcccgttttgtataagtacctggagcttcaggataaagcagttactgtggacgaatatcaaaaggcccaagaaaagtggatgaaagagaaggaagagtctaacaaaaaggcacaggaagacttggctaagcacgtaaaatgaaagctt | SGSGSSTKGEKTFSYIYETDPDNLNYLTTAKAATANITSNVVDGLLENDRYGNFVPSMAE  DWSVSKDGLTYTYTIRKDAKWYTSEGEEYAAVKAQDFVTGLKYAADKKSDALYLVQESIK  GLDAYVKGEIKDFSQVGIKALDEQTVQYTLNKPESFWNSKTTMGVLAPVNEEFLNSKGDD  FAKATDPSSLLYNGPYLLKSIVTKSSVEFAKNPNYWDKDNVHVDKVKLSFWDGQDTSKPA  ENFKDGSLTAARLYPTSASFAELEKSMKDNIVYTQQDSITYLVGTNIDRQSYKYTSKTSD  EQKASTKKALLNKDFRQAIAFGFDRTAYASQLNGQTGASKILRNLFVPPTFVQADGKNFG  DMVKEKLVTYGDEWKDVNLADSQDGLYNPEKAKAEFAKAKSALQAEGVQFPIHLDMPVDQTATTKVQRVQSMKQSLEATLGADNVIIDIQQLQKDEVNNITYFAENAAGEDWDLSDNVGWGPDFADPSTYLDIIKPSVGESTKTYLGFDSGEDNVAAKKVGLYDYEKLVTEAGDETTDVAKRYDKYAAAQAWLTDSALIIPTTSRTGRPILSKMVPFTIPFALSGNKGTSEPVLYKYLEL  QDKAVTVDEYQKAQEKWMKEKEESNKKAQEDLAKHVK |
| **PcsB (SP_2216)** | ggtaccggtgaaaccacggacgataaaatcgcggcacaggacaataaaattagtaatctgacggcacagcaacaggaagcccaaaagcaagtggatcaaattcaagagcaagtgtccgcaattcaggcagagcagagcaatttgcaggccgagaatgatcgcctgcaagctgagtccaagaaacttgagggagaaatcactgaactttccaagaacatcgtgtctcgcaaccagtcattagaaaagcaagcccgttcggcgcaaaccaatggcgctgtgacatcatacatcaataccatcgttaattctaaatctatcactgaagccatttcccgcgtggcagcaatgtccgaaatcgtatcagcaaataacaagatgcttgagcaacagaaggcggataagaaggctatctctgaaaaacaagtagcaaacaatgacgcaatcaacacagtcattgcgaaccagcaaaagttagccgatgacgcgcaagccttaactacgaagcaagctgagctgaaagccgccgaactgagtttggcggctgaaaaagctacggcagagggagaaaaagctagtttattagagcaaaaagccgctgcggaagcggaagcacgcgcagcggctgtggctgaggcggcttataaagagaagcgtgcttctcaacaacaatcggtactggcttctgctaataccaatcttactgcacaagtgcaagcagtatcagaaagtgcggcagctccagtgcgcgcgaaggtacgcccgacctacagtacaaatgcgagttcatatccaatcggggaatgcacttggggagttaagaccttagcaccgtgggcaggcgattactgggggaatggtgcccaatgggctacgagcgccgcagcggcgggctttcgcactggttcaacgccccaggttggggcgattgcatgttggaacgacggaggatacggtcacgtcgccgtcgtaacagcggttgaatcgacaacccgtattcaggtgtctgagagtaattatgccggtaaccgcacgattggcaatcatcgtggatggttcaaccccaccacaacgagtgaaggcttcgtcacctatatctacgcggattgaaagctt | ETTDDKIAAQDNKISNLTAQQQEAQKQVDQIQEQVSAIQAEQSNLQAENDRLQAESKKLEGEITELSKNIVSRNQSLEKQARSAQTNGAVTSYINTIVNSKSITEAISRVAAMSEIVSAN  NKMLEQQKADKKAISEKQVANNDAINTVIANQQKLADDAQALTTKQAELKAAELSLAAEKATAEGEKASLLEQKAAAEAEARAAAVAEAAYKEKRASQQQSVLASANTNLTAQVQAVSESAAAPVRAKVRPTYSTNASSYPIGECTWGVKTLAPWAGDYWGNGAQWATSAAAAGFRTGSTPQVGAIACWNDGGYGHVAVVTAVESTTRIQVSESNYAGNRTIGNHRGWFNPTTTSEGFVTYIYAD |
| **AdcAII (SP_1002)** | Ggtaccggtcagactggaaagggtatgaaaatcgtaacttctttttatccaatttacgctatggtcaaggaggtatccggtgatctgaacgatgtgcgtatgatccagagcagtagtggaatccattcctttgaaccatccgccaatgatattgccgccatctacgatgcagacgtgttcgtttaccattcccatacgctggaatcctgggcgggcagtttagatccgaatctgaagaaaagcaaggttaaggtacttgaggcttccgagggtatgactctggaacgtgtacccggattggaggacgtcgaggcgggcgacggcgttgacgagaagaccctttacgatcctcacacctggttggaccctgaaaaggcaggtgaggaggcccagatcatcgctgataaactgagtgaggtcgattctgaacataaggagacgtaccagaagaatgcccaagcctttatcaagaaagctcaggagttaaccaaaaaattccaaccgaagtttgaaaaggcaacgcagaagactttcgttacacagcacaccgcgttctcctatctggctaagcgtttcggattaaaccagttgggtattgcagggattagcccggagcaagagccatcgccgcgccaacttacagagatccaggagtttgttaagacatataaggtaaaaaccatttttacggaatcgaatgccagctcgaaggtagcagagacgttagtaaagtctacaggcgtcggactgaaaactctgaatcctctggagtcagatcctcaaaacgataagacttacttggagaatttggaagaaaacatgtccattcttgccgaggaattaaagtgaaagctt | QTGKGMKIVTSFYPIYAMVKEVSGDLNDVRMIQSSSGIHSFEPSANDIAAIYDADVFVYH  SHTLESWAGSLDPNLKKSKVKVLEASEGMTLERVPGLEDVEAGDGVDEKTLYDPHTWLDPEKAGEEAQIIADKLSEVDSEHKETYQKNAQAFIKKAQELTKKFQPKFEKATQKTFVTQHT  AFSYLAKRFGLNQLGIAGISPEQEPSPRQLTEIQEFVKTYKVKTIFTESNASSKVAETLVKSTGVGLKTLNPLESDPQNDKTYLENLEENMSILAEELK |
| **PrtA (SP_0641)** | ggtaccggagacacatctagctctgaagatgctttaaacatctctgataaagaaaaagtagcagaaaataaagagaaacatgaaaatatccatagtgctatggaaacttcacaggattttaaagagaagaaaacagcagtcattaaggaaaaagaagttgttagtaaaaatcctgtgatagacaataacactagcaatgaagaagcaaaaatcaaagaagaaaattccaataaatcccaaggagattatacggactcatttgtgaataaaaacacagaaaatcccaaaaaagaagataaagttgtctatattgctgaatttaaagataaagaatctggagaaaaagcaatcaaggaactatccagtcttaagaatacaaaagttttatatacttatgatagaatttttaacggtagtgccatagaaacaactccagataacttggacaaaattaaacaaatagaaggtatttcatcggttgaaagggcacaaaaagtccaacccatgatgaatcatgccagaaaggaaattggagttgaggaagctattgattacctaaagtctatcaatgctccgtttgggaaaaattttgatggtagaggtatggtcatttcaaatatcgatactggaacagattatagacataaggctatgagaatcgatgatgatgccaaagcctcaatgagatttaaaaaagaagacttaaaaggcactgataaaaattattggttgagtgataaaatccctcatgcgttcaattattataatggtggcaaaatcactgtagaaaaatatgatgatggaagggattattttgacccacatgggatgcatattgcagggattcttgctggaaatgatactgaacaagacatcaaaaactttaacggcatagatggaattgcacctaatgcacaaattttctcttacaaaatgtattctgacgcaggatctgggtttgcgggtgatgaaacaatgtttcatgctattgaagattctatcaaacacaacgttgatgttgtttcggtatcatctggttttacaggatgaaagctt | DTSSSEDALNISDKEKVAENKEKHENIHSAMETSQDFKEKKTAVIKEKEVVSKNPVIDNN  TSNEEAKIKEENSNKSQGDYTDSFVNKNTENPKKEDKVVYIAEFKDKESGEKAIKELSSL  KNTKVLYTYDRIFNGSAIETTPDNLDKIKQIEGISSVERAQKVQPMMNHARKEIGVEEAI  DYLKSINAPFGKNFDGRGMVISNIDTGTDYRHKAMRIDDDAKASMRFKKEDLKGTDKNYWLSDKIPHAFNYYNGGKITVEKYDDGRDYFDPHGMHIAGILAGNDTEQDIKNFNGIDGIAPNAQIFSYKMYSDAGSGFAGDETMFHAIEDSIKHNVDVVSVSSGFTG |
| **PsaA (SP_1650)** | ggtaccggtgcttccggcaaaaaggatactacaagcggtcaaaaattaaaagtggttgcaactaattctatcatcgcagacatcactaaaaatatcgctggcgataagattgatcttcatagtatcgttcctattgggcaagatccgcacgaatatgaacctttacctgaggacgtaaaaaagacgtccgaggcgaatctgattttctataacgggattaacctggaaactggtggcaatgcttggtttacaaagttagtcgagaatgctaagaaaacggagaataaagactactttgccgtcagtgacggagttgatgtaatttatttagagggtcaaaacgaaaaaggcaaggaggaccctcacgcctggttaaacttagagaatggcattatctttgctaaaaacatcgctaagcaattaagtgccaaagatccaaataacaaagagttctacgaaaagaatctgaaagagtatacggacaaactggataaacttgataaggagtcgaaagataaatttaacaagattcccgcagagaaaaaattaattgtgacgtccgagggggcttttaagtatttcagtaaagcctatggtgtgccaagcgcctacatttgggaaatcaacaccgaggaagaaggcacccccgagcaaattaagacattggtagaaaaattacgtcagactaaagttccgagtttgtttgtggagtcatcggttgatgaccgcccaatgaagactgtgagccaagatactaacatccctatctatgcccaaatcttcaccgattcgattgctgagcaaggcaaggaaggagactcttactacagtatgatgaaatacaacctggataagatcgctgagggcttagctaaatgaaagctt | ASGKKDTTSGQKLKVVATNSIIADITKNIAGDKIDLHSIVPIGQDPHEYEPLPEDVKKTS  EADLIFYNGINLETGGNAWFTKLVENAKKTENKDYFAVSDGVDVIYLEGQNEKGKEDPHA  WLNLENGIIFAKNIAKQLSAKDPNNKEFYEKNLKEYTDKLDKLDKESKDKFNKIPAEKKL  IVTSEGAFKYFSKAYGVPSAYIWEINTEEEGTPEQIKTLVEKLRQTKVPSLFVESSVDDR  PMKTVSQDTNIPIYAQIFTDSIAEQGKEGDSYYSMMKYNLDKIAEGLAK |
