# Supplementary material for: Exploring metal availability in the natural niche of Streptococcus pneumoniae to discover potential vaccine antigens
Source: Virulence. 2020 Oct 5;11(1):1310–28. doi: 10.1080/21505594.2020.1825908 (PMC7550026; doi:10.1080/21505594.2020.1825908)
Supplement: Supplemental Material [file KVIR_A_1825908_SM6912.zip › Table_S9.docx]

**Table S9. Primer sets used for *in vivo* gene expression analysis of *S. pneumoniae***

| Target gene | qPCR primer sequence |
| --- | --- |
| SP_0268_spuA_F | ACCCGAAAGGACTTGAATGG |
| SP_0268_spuA_R | AGTCTGGTTTCTCTTCTGCAG |
| SP_1069_tprX_F | TGCATCAGGATTTCCAACGG |
| SP_1069_tprX_R | CCAAACCACCTACTTCTACC |
| SP_0149_metQ_F | AACGGAGAAATCGCTGTACC |
| SP_0149_metQ_R | GAGCAGTTCCAGAAACATCC |
| SP_0749_livJ_F | CAGTCAACACTGCTGGTAAC |
| SP_0749_livJ_R | TTCAGCTGTTCCGTATGCAG |
| SP_0366_aliA_F | TTGCCGATCCATCAACCTAC |
| SP_0366_aliA_R | GCAGCTACATTATCTTCCCC |
| SP_2216_pcsB_F | CAAGCTCGTAGTGCTCAAAC |
| SP_2216_pcsB_R | TCACTCATTGCAGCAACACG |
| SP_1002_adcAII_F | AATGACCTTGGAACGTGTCC |
| SP_1002_adcAII_R | TCAGGATCTAGCCATGTGTG |
| SP_0641_prtA_F | TGATGAGATTGCAAATGAGG |
| SP_0641_prtA_R | TTTCTTCCTTGACCCTCACC |
| sp_1650_psaA_F | GCTACAAACTCAATCATCGCTGAT |
| sp_1650_psaA_R | CGTCTTCAGGAAGTGGTTCGTAT |
